# High-affinity ligands of the colchicine domain in tubulin based on a structure-guided design

**DOI:** 10.1038/s41598-018-22382-x

**Published:** 2018-03-09

**Authors:** Oskía Bueno, Juan Estévez Gallego, Solange Martins, Andrea E. Prota, Federico Gago, Asier Gómez-SanJuan, María-José Camarasa, Isabel Barasoain, Michel O. Steinmetz, J. Fernando Díaz, María-Jesús Pérez-Pérez, Sandra Liekens, Eva-María Priego

**Affiliations:** 1 Instituto de Química Médica (IQM,CSIC), Juan de la Cierva 3, 28006 Madrid, Spain; 2 Centro de Investigaciones Biológicas (CIB,CSIC), Ramiro de Maeztu 9, 28040 Madrid, Spain; 3 Rega Institute for Medical Research, KU Leuven, Herestraat 49, B-3000 Leuven, Belgium; 4 Laboratory of Biomolecular Research, Division of Biology and Chemistry, Paul Scherrer Institut, 5232 Villigen, Switzerland; 5 Department of Biomedical Sciences (Unidad Asociada IQM,CSIC) and Instituto de Investigación Quimica “Andrés M. del Río” (IQAR), University of Alcalá, Unidad Asociada CSIC, 28805 Alcalá de Henares, Madrid, Spain; 6 University of Basel, Biozentrum, CH-4056 Basel Switzerland

## Abstract

Microtubule-targeting agents that bind at the colchicine-site of tubulin are of particular interest in antitumoral therapy due to their dual mechanism of action as antimitotics and vascular disrupting agents. Cyclohexanediones derivatives have been described as a new family of colchicine-domain binders with an association constant to tubulin similar to that of colchicine. Here, the high-resolution structures of tubulin in complex with cyclohexanediones TUB015 and TUB075 were solved by X-ray crystallography. A detailed analysis of the tubulin-TUB075 interaction by means of computational affinity maps allowed the identification of two additional regions at the binding site that were addressed with the design and synthesis of a new series of cyclohexanediones with a distal 2-substituted benzofurane. These new compounds showed potent antiproliferative activity with IC_50_ values in the nM range, arrested cell cycle progression at the G_2_/M phase and induced apoptosis at sub μM concentrations. Moreover, they caused the destruction of a preformed vascular network i*n vitro* and inhibited the migration of endothelial cells at non-toxic concentrations. Finally, these compounds displayed high affinity for tubulin as substantiated by a *K*_*b*_ value of 2.87 × 10^8^ M^−1^ which, to the best of our knowledge, represents the highest binding constant measured to date for a colchicine-domain ligand.

## Introduction

Microtubules are cytoskeletal fibers, composed of αβ-tubulin heterodimers, which play a major role in mitosis, maintenance of cell shape and structure, and cell motility^1^. Their formation is highly dynamic involving polymerization/depolymerization of the αβ-tubulin heterodimers, a process that is crucial for cell survival^2,3^. Compounds that alter this equilibrium of assembly/disassembly between heterodimers induce, among other events, cell cycle arrest and apoptosis^4,5^.

**Figure 1:**
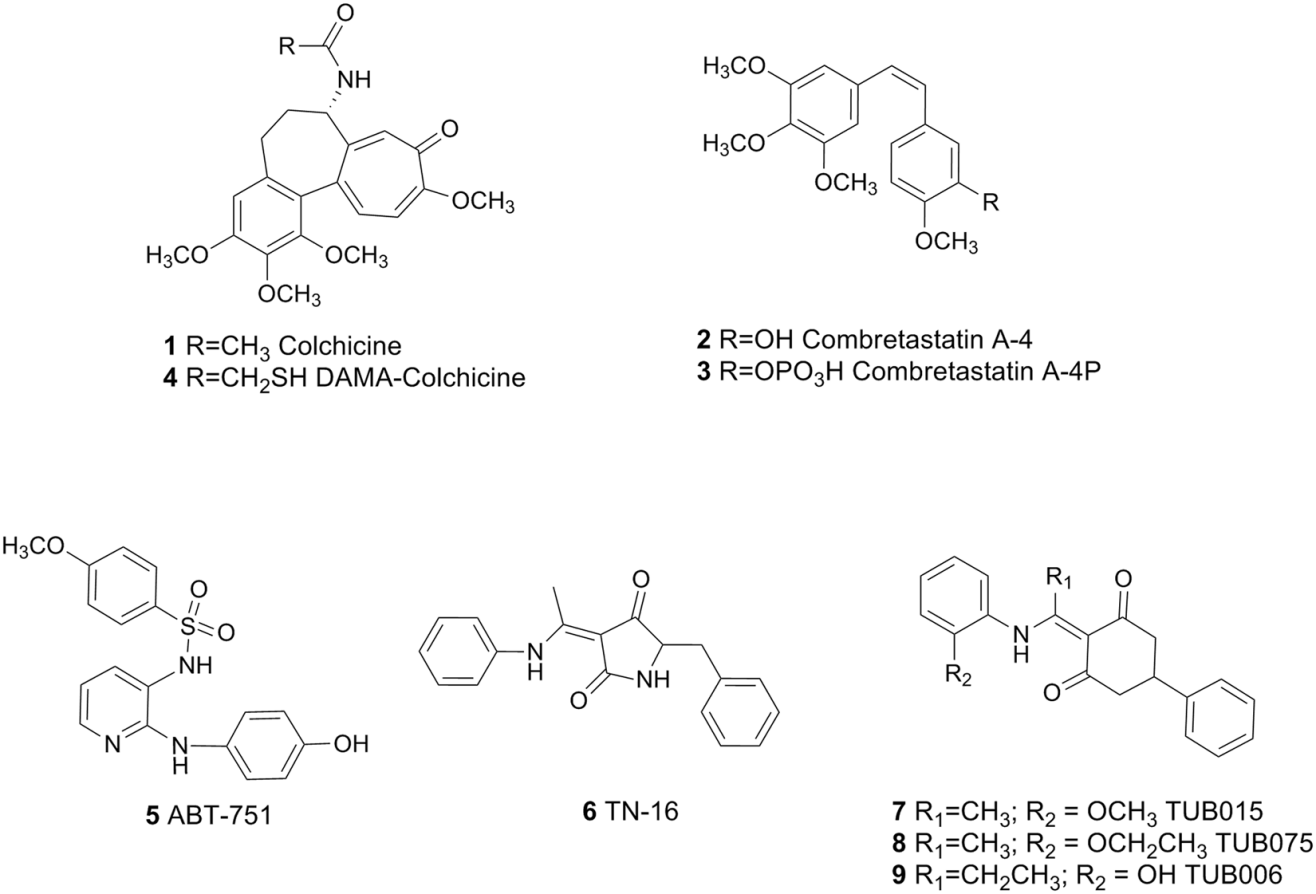
Representative colchicine-site ligands and selected cyclohexanedione derivatives.

Among microtubule-targeting agents (MTAs), colchicine site binders are being deeply investigated^6^. Colchicine (**1**, Fig. 1), isolated from the seeds and bulbs of *Colchicum autumnale*, is an old drug that not only allowed the isolation and characterization of tubulin^7,8^ and that has some therapeutic applications such as treatment of gout or familiar Mediterranean fever^9^. However, its toxicity halted its use as an anticancer agent. Interestingly, combretastatin A-4 (**2**, CA-4) a natural product isolated from *Combretum caffum*^10^, which also binds at the colchicine site^11^, is being evaluated in the clinic for oncology applications. Besides its antimitotic activity, CA-4 has shown vascular disrupting capacity^12^. It is well documented that the differences between physiological and intratumoral vessels are significant enough to allow a selective therapeutic action on the latter^13,14^. Consequently, CA-4 and its prodrug CA-4P (**3**, Fosbretabulin), represent the prototype vascular disrupting agents (VDA) among MTAs^15,16^. However, the chemical instability and low solubility of CA-4 has promoted the search and exploration of other chemical classes able to bind at the colchicine site in tubulin that combine antimitotic and antivascular properties, as recently reviewed^17–21^.

Atomic details of the colchicine-binding site were published in 2004, when Ravelli *et al*.^22^ solved the structure of αβ-tubulin with the stathmin-like domain of the RB3 protein in complex with *N*-deacetyl-*N*-(2-mercaptoacetyl)colchicine (**4**, DAMA-colchicine, PDB ID: 1SA0). In 2009, besides the unliganded tubulin, complexes with structurally different ligands such as the sulfonamide ABT715 (**5**, PDB ID: 3HKC) or TN-16 (**6**, PDB ID: 3HKD) were reported, and the definition of the colchicine-binding site was expanded to that of a “colchicine-binding domain”^23^. More recently, higher-resolution structures (<2.5 Å) have been obtained using tubulin tyrosine ligase (TTL)^24,25^, a well-known enzyme involved in the post-translational modifications of the C-terminal tail of tubulin. Therefore, crystals containing two heterodimers of tubulin (T_2_), the stathmin-like domain of RB3 (R) and TTL were solved at 2.6–1.8 Å resolution^26,27^. In the last two to three years, the number of solved complexes has increased significantly^11,28–30^. This information could facilitate the structure-based optimization of the described ligands. However, to the best of our knowledge, such ligand optimization has not been reported.

We have demonstrated that compounds with a cyclohexanedione central scaffold are able to bind tubulin at the colchicine site with an association constant (K_a_ 1.3 × 10^7^ M^−1^) similar to that of colchicine^31,32^. These compounds, represented by the prototypes **7** (TUB015) and **8** (TUB075), showed potent antiproliferative activity against tumor and endothelial cells (IC_50_ around 0.08–0.19 μM), causing a dose-dependent increase in the G_2_/M phase and inducing apoptosis. The initial hit (**9**, TUB006), which was 100-fold less active both in tubulin binding and in antiproliferative assays, was identified through a virtual screening campaign using the 3D-shape of TN-16 (**6**) in complex with tubulin (PDB ID: 3HKD) as the query^31^. Thus, these cyclohexanediones were expected to bind similarly to TN-16 in the colchicine-binding domain. To confirm this and to dissect the critical interactions between our compounds and the colchicine-binding site, the X-ray crystal structures of both T_2_R-TTL-TUB075 and T_2_R-TTL-TUB015 complexes have been solved. Based on this detailed information and guided by affinity maps generated with the cGRILL software^33,34^, a structure-based synthesis of new cyclohexanedione derivatives has been accomplished. The binding to tubulin and the antiproliferative, antimigratory and vascular disrupting properties of this new series of compounds have also been evaluated.

## Results and Discussion

### Crystal structure of the tubulin-TUB015 and tubulin–TUB075 complexes

**Figure 2:**
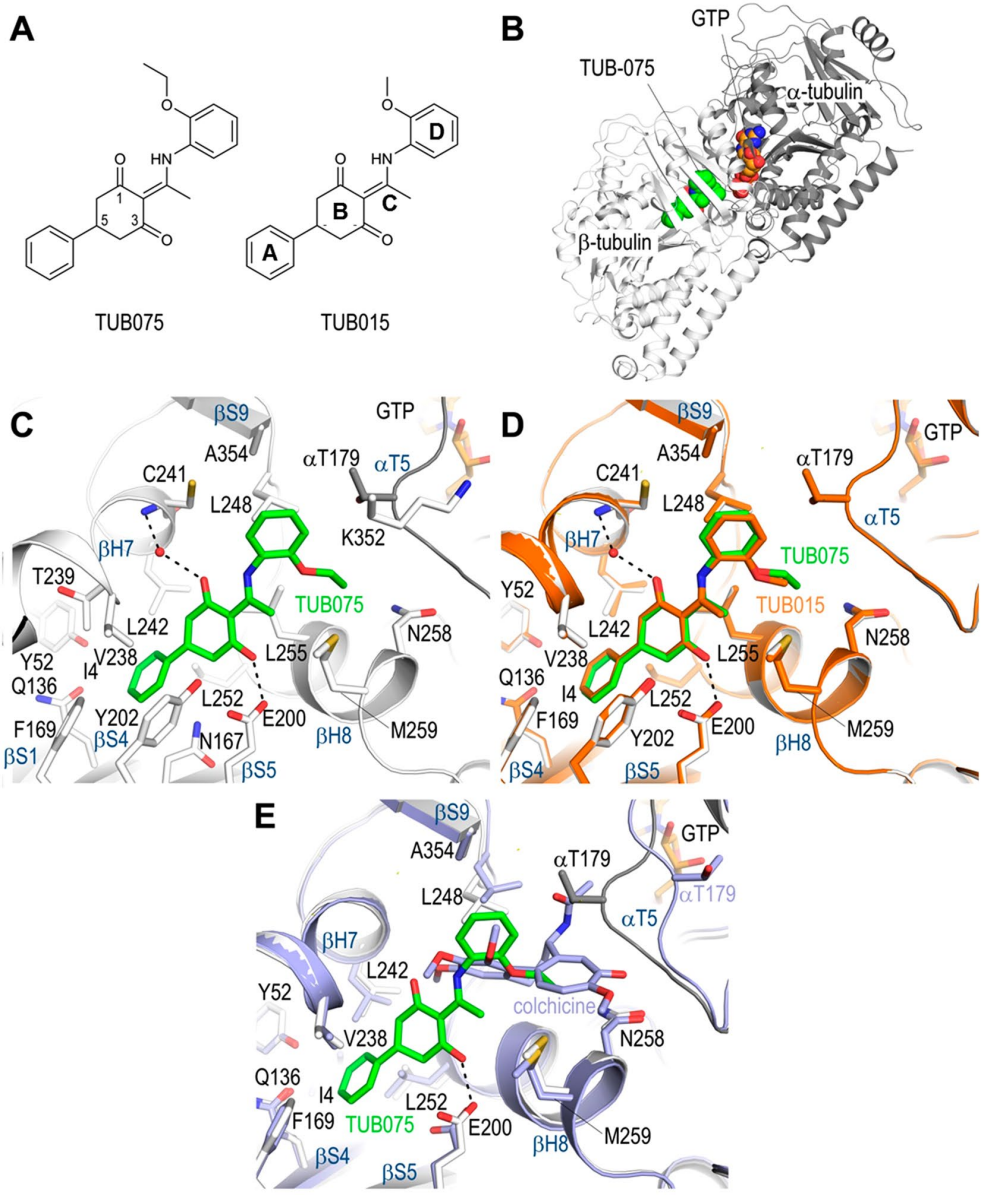
Crystal structures of the tubulin-TUB075 and TUB015 complexes. Overall structure of the T_2_R-TTL-TUB075 complex. (**A**) Chemical structures and defined fragments of TUB075 and TUB015. (**B**) Overall view of the T_2_R-TTL-TUB075 complex structure. The α-tubulin and β-tubulin chains are in dark and light grey ribbon representation, respectively. The tubulin-bound TUB075 and the GTP molecules are represented as green and orange spheres, respectively. (**C**) Close-up view of the interaction network observed between TUB075 (green) and tubulin (gray). Interacting residues of tubulin are shown in stick representation and are labeled. Oxygen and nitrogen atoms are colored red and blue, respectively; carbon atoms are in green (TUB075) or gray (tubulin). Hydrogen bonds are depicted as black dashed lines. Secondary structural elements of tubulin are labeled in blue. For simplicity, only α-tubulin residues are indicated with an α. (**D**) The same close-up view as in (**C**) with the superimposed TUB015 (orange) structure. (**E**) Superimpositions of the tubulin-TUB075 (green/grey) and tubulin-colchicine (cyan; PDB ID 4O2B; rmsd of 0.395 Å over 422 Cα-atoms) complex structures in the same orientation and representation as in panel (C). The structures were superimposed onto their β_1_-tubulin chains by secondary structure matching in Coot.

To obtain a detailed description of how TUB015 (**7**)^31^ and TUB075 (**8**)^32^ interact with tubulin and to establish a firm basis for the design of TUB-derivatives with improved affinities, we solved the structures of tubulin in complex with TUB015 and TUB075 (Fig. 2A) by X-ray crystallography. To this end, we soaked the ligands into crystals formed by a protein complex composed of two bovine brain αβ-tubulin heterodimers, the rat stathmin-like protein RB3 and chicken tubulin tyrosine ligase (T_2_R-TTL)^24,25^. Using this approach, both the tubulin-TUB075 and tubulin-TUB015 complex structures were determined to 2.15 and 2.1 Å resolution, respectively (Figs S1 and 2B; Table S1). Comparison of the overall structure of tubulin in both the T_2_R-TTL-TUB075 and the T_2_R-TTL-TUB015 complexes with the one obtained in the absence of any ligand^25^ revealed a good superimposition (rmsd_T2R-TTL-TUB075_ of 0.37 Å over 1925 C_α_-atoms; rmsd_T2R-TTL-TUB015_ of 0.34 Å over 1921 C_α_-atoms), suggesting that binding of both ligands does not affect the overall conformation of tubulin. Both ligands bind to the colchicine domain of tubulin, which is formed by residues of strands S8, S9 and S10, loop T7, and helices H7 and H8 of β-tubulin, and loop T5 of α-tubulin (Fig. 2C). The differences between TUBs and colchicine binding will be detailed in the next paragraphs.

In both the tubulin-TUB075 and tubulin-TUB015 complexes, the 5-phenyl moiety of the cyclohexanedione ring (ring A in Fig. 2A) is buried in the pocket shaped by the side chains of βIle4, βTyr52, βGln136, βAsn167, βPhe169, βGlu200, βTyr202, βVal238, βThr239, βLeu242, and βLeu252 (Fig. 2C,D). Furthermore, the cyclohexanedione ring (ring B in Fig. 2A) is stacked between βTyr202 and βLeu255, and is further stabilized by two polar contacts, as follows. In both molecules one direct hydrogen bond is formed between the C3-carbonyl of the ligand and the side chain of βGlu200. A second hydrogen bond, water mediated, is formed by the C1-carbonyl to the main chain amide of βCys241. The water molecule is well defined in the TUB015 complex, and partially occupied in the tubulin-TUB075 complex. In agreement with the suggestion recently put forward by Guzmán-OCampo *et al*.^35^ that, in the tubulin-nocodazole complex, the side-chain of βGlu200 is likely to exist as a carboxylic acid rather than as a carboxylate, we considered the C-3 carbonyl oxygen to be engaged in a hydrogen bonding interaction with this moiety, given the suitable distance and angle geometries. The 2-ethoxy/2-methoxy aniline moiety (ring D in Fig. 2A) of both TUB compounds is solely stabilized by hydrophobic contacts to βLeu248, βMet259, βAsn258, βAla317, βLys352, βala354, and to the T5 loop residue αThr179. This binding mode is maintained in both the occupied binding sites in our crystal system (TUB075: RMSD_chain D onto chain B_ 0.37 Å over 420 C_α_-atoms; TUB015: RMSD _chain D onto chain B_ 0.37 Å over 420 Cα-atoms).

Compared to the crystal structure of the tubulin-colchicine complex (PDB ID 4O2B; Fig. 2E) ^26^, there are certain differences that need to be highlighted. Probably the most significant difference is the change in the αT5 loop, which is flipped out in the colchicine structure to avoid clashes with the acetamide moiety of the colchicine molecule while in the TUB complexes this loop is closing the binding site. Also the side chain of βLeu255 is blocking the access to the most internal subsite in the colchicine complex, while the binding of the TUB compounds causes a flip in this side chain to access this internal subpocket. Altogether, little overlap is observed among the tubulin-bound TUB and colchicine molecules (Fig. 2E).

According to the structural interaction fingerprints (SIFTs) reported by Massaroti *et al*.^36^, the colchicine domain can be divided into 3 zones: zone 1, located at the α-subunit interface; zone 2, that is the main zone located in the β-subunit which accommodates most of the structure of the ligands, and zone 3, an accessory pocket buried deeper in the β-subunit. For binding, TUB015 and TUB075 explore zones 2 and 3, as reported for other ligands, particularly TN-16, but the methoxy/ethoxy substituent points towards zone 1.

Notably, the binding of TUB015 and TUB075 is in full agreement with the structure-activity relationships (SAR) previously established for this family of compounds^31,32^ which are graphically summarized in Supplementary Fig. S2.

### Design of new ligands based on affinity maps

**Figure 3:**
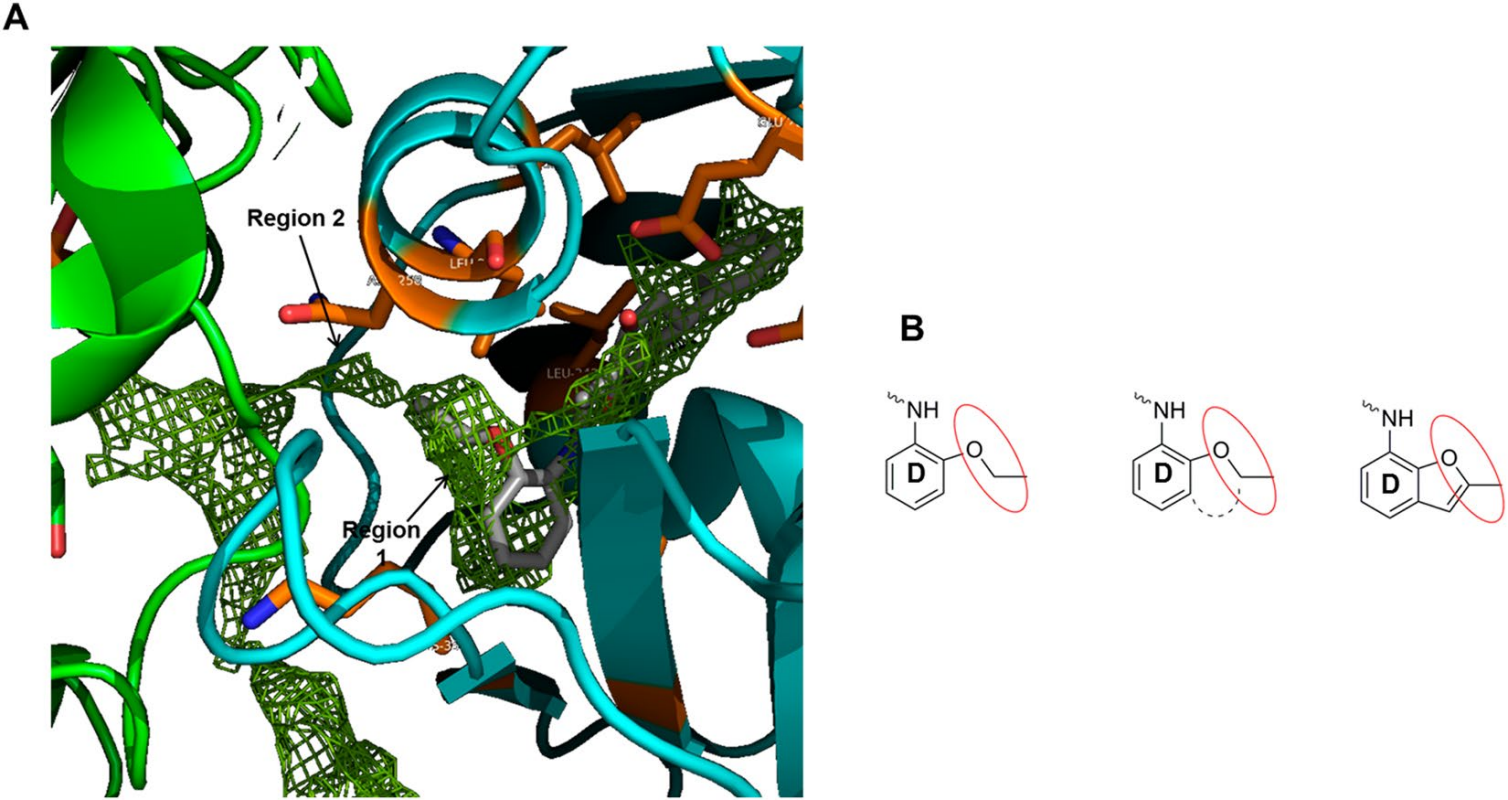
Affinity maps and benzofurane ring design. (**A**) Contour maps generated by cGRILL for a hydrophobic probe in a ligand-free colchicine binding site obtained from the tubulin-TUB075 complex. α-Tubulin in green, β-tubulin in cyan and TUB075 in grey sticks. (**B**) Benzofurane ring design to occupy region 1.

To better analyze the binding of TUBs to tubulin, affinity maps were generated using our in-house computational tool cGRILL^33,34^, which relies on GRID functions as developed by Goodford *et al*.^37^. By applying different chemical probes, insight can be gained about both favorable binding interactions and forbidden areas. Using the lipophilic probe (CH_3_ + no possibility of forming hydrogen bonds, akin to GRID’s DRY probe) on the TUB075-tubulin complex, two new unexplored regions were identified in the proximity of the aniline moiety (ring D) as suitable for binding a short hydrocarbon chain (Fig. 3A). Region 1 covers the ethoxy substituent at position 2 and the adjacent position 3 of the aromatic ring. This zone could be occupied by a ring fused to positions 2 and 3 of the phenyl ring resulting in a 2-methylbenzofurane, as depicted in Fig. 3B. This substitution, besides keeping the existing favorable interactions of TUB075, should cover region 1 identified by cGRILL and fix the conformation of the ethoxy substituent of TUB075. The second region, with a tunnel shape, extends from the ethoxy substituent towards the αβ-tubulin interface, suggesting the 2-methyl substituent of the benzofurane could be enlarged to occupy this tunnel.

### Synthesis of new derivatives

**Figure 4:**
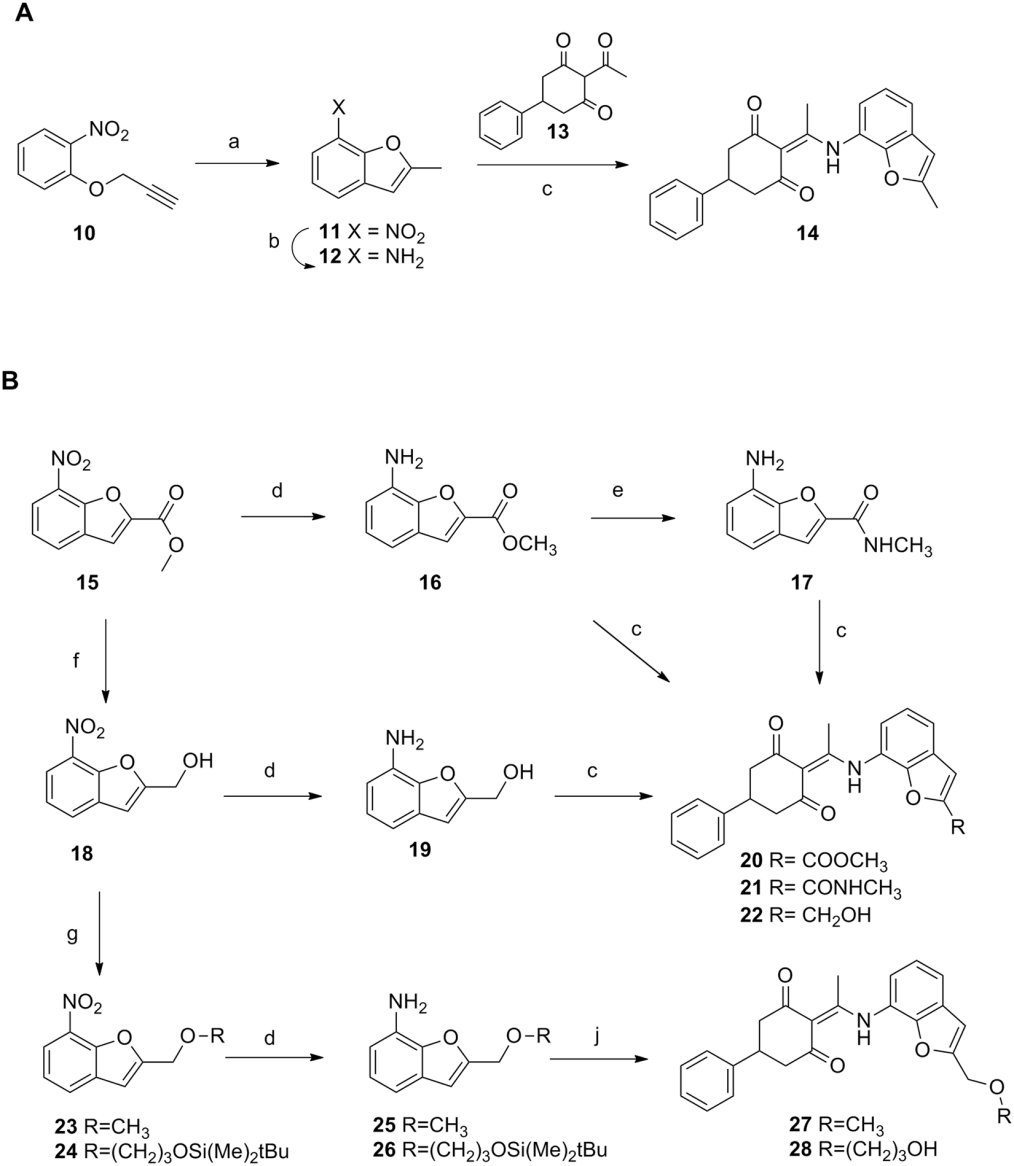
Synthesis of the benzofurane derivatives. Reagents and conditions: (a) PEG-500, 215 °C, 90 min; (b) SnCl_2_, HCl, MeOH, 75 °C, 30 min; (c) **13**, toluene, 4 Å molecular sieves, pressure tube, 110 °C, overnight. (d) H_2_, 5% Pt/S, AcOEt, 30 psi, rt, 2–8 h.; (e) NH_2_CH_3_, NH_4_Cl, MeOH, MW, 60 °C, 3 h; (f) DIBAL, Et_2_O, rt, 6 h; (g) **13**, toluene, 4 Å molecular sieves, pressure tube, 110 °C, overnight. (h) For **23**: MeI, NaH, anh. DMF, rt, 2 h; for **24**: (3-bromopropoxy)(*tert*-butyl)dimethylsilane, 50% NaOH, TBABr, THF, 60 °C, 4 h; (j) for **27**: **13**, toluene, 4 Å molecular sieves, pressure tube, 110 °C, overnight; (j) for **28**: (i) **13**, toluene, 4 Å molecular sieves, pressure tube, 110 °C, overnight, (ii) TFA, CH_2_Cl_2_, rt, 1 h.

The synthesis of the 2-methylbenzofurane derivative was addressed as depicted in Fig. 4A. Intramolecular cyclization of the propyne derivative **10**^38^ at high temperature^39^ afforded the nitro derivative **11**^40^ that was hydrogenated to provide the amino derivative **12**^41^. Condensation of this amine with 2-acetyl-5-phenylcyclohexane-1,3-dione (**13**)^31^ in refluxing toluene afforded the target compound **14** in 74% yield.

To include an additional functionalization at position 2 of the benzofurane that should allow elongation of the TUB structures towards the tunnel identified as region 2 in the cGRILL maps, new derivatives were envisioned where the methyl group of **14** was replaced by an ester, an amide or an alcohol. The 7-nitrobenzofuran-2-methyl ester (**15**)^42^ (Fig. 4B) was synthesized following a described procedure^42^ and further hydrogenated in the presence of 5% Pt/S to afford the corresponding amino derivative **16**^43^. Reaction of the ester **16** with methylamine quantitatively provided the amide **17**. Alternatively, reduction of the ester **15** with DIBAL afforded the alcohol **18** that was hydrogenated to provide the amino derivative **19**. Condensation of the amines **16**, **17** or **19** with the cyclohexanedione **13** afforded **20**, **21** and **22** in 43, 76 and 79% yield, respectively. Interestingly, only the alcohol derivative **22** kept antiproliferative activity similar to that of the 2-methylbenzofurane **14** whiles the ester **20** and the amide **21** were significantly less active.

Additionally, reaction of the alcohol **18** (Fig. 4B) with methyl iodide in the presence of NaH afforded the methoxy derivative **23** in quantitative yield. Similarly, reaction of **18** with (3-bromopropoxy)(*tert*-butyl)dimethylsilane^44^ under basic conditions afforded **24** in 85% yield. Reduction of the nitro group in **23** and **24** by hydrogenation in the presence 5% Pt/S afforded the amines **25** and **26** in 84 and 71% yields, respectively. Condensation of **25** with the acylcyclohexanedione **13** provided the methoxy derivative **27** in 82% yield. Similarly, reaction of the amine **26** with **13** followed by reaction with TFA to remove the silyl protecting group afforded the alcohol **28** in 65% yield.

### Antiproliferative activity

The anti-proliferative activity of the synthesized benzofurane derivatives was studied in three human cancer cell lines [breast carcinoma (MDA-MB-231), lymphoblastic leukemia (CEM) and cervical carcinoma (HeLa) cells] and one endothelial cell line [human microvascular endothelial cells (HMEC-1)]. Data are collected in Table 1 and expressed as IC_50_ (50% inhibitory concentration) values, that is, the concentrations at which the compounds reduce cell proliferation by 50%. As reference compounds, colchicine (**1**) and CA-4P (**3**) are included. Our previous hit compound **8** has also been included for comparative purposes.

**Table 1:** Anti-proliferative activity of the benzofurane derivatives in endothelial and tumor cell lines and binding constants for αβ-tubulin.

Comp.	Tumor cells IC_50_ (μM)^a^			Endothelial cells IC_50_ (μM)^a^	K_b_ (M^−1^)
	MDA-MB-231	CEM	HeLa	HMEC-1	
**8**	0.068 ± 0.005	0.19 ± 0.01	0.18 ± 0.0	0.10 ± 0.02	(13 ± 2) × 10^6(b)^
**14**	0.053 ± 0.006	0.038 ± 0.001	0.16 ± 0.01	0.065 ± 0.003	(89.5 ± 3.6) × 10^6(b)^
**20**	0.17 ± 0.01	0.64 ± 0.36	0.89 ± 0.03	1.6 ± 0.6	(1.57 ± 0.35) × 10^6(b)^
**21**	1.5 ± 0.6	5.5 ± 1.7	12 ± 6	14 ± 11	(0.07 ± 0.01) × 10^6(b)^
**22**	0.015 ± 0.002	0.040 ± 0.002	0.14 ± 0.04	0.15 ± 0.05	(91.0 ± 10.1) × 10^6(b)^
**27**	0.009 ± 0.001	0.031 ± 0.002	0.030 ± 0.002	0.016 ± 0.002	(287 ± 106) × 10^6(b)^
**28**	0.012 ± 0.005	0.051 ± 0.018	0.034 ± 0.003	0.038 ± 0.003	(65.1 ± 6.80) × 10^6(b)^
Colchicine	0.007 ± 0.003	0.013 ± 0.0004	0.0087 ± 0.0001	0.0038 ± 0.0011	11.6 × 10^6(c)^^63^
CA-4P	0.0021 ± 0.0003	0.011 ± 0.001	0.013 ± 0.001	0.0029 ± 0.0001	
Podophillotoxin	—	—	—	—	1.8 × 10^6^ ^64^
R-PT	—	—	—	—	3.2 × 10^6 ^^46^

^a^IC_50_ (50% inhibitory concentration) is given as the mean ± SD of three independent experiments.

^b^Mean value of three experiments ± SD.

^c^At 37 °C.

Compound **14** provided similar or lower IC_50_ values than the reference compound **8** suggesting that the incorporation of the fused ring is beneficial for the antiproliferative activity. Incorporation of a methyl ester or methylamide functionality at position 2 of the benzofurane (compounds **20** and **21**) had a negative impact on the cytostatic activity, while compound **22** with a hydroxymethyl group provided IC_50_ values comparable to those of the methyl derivative **14**. Interestingly further substitution of the hydroxymethyl group as in compounds **27** and **28** resulted in IC_50_ values ranging from 0.009 to 0.051 μM. Thus, compounds **27** and **28** emerged as the most potent antiproliferative compounds among cyclohexanedione derivatives.

One of the most relevant causes of tumor resistance to chemotherapy is the overexpression of membrane pumps which remove the chemotherapeutic agents from the treated cells^45^. To evaluate the effect of the new chemotype on resistant cells, the most representative compounds were tested against A2780 and A2780AD (MDR overexpressing P-glycoprotein) human ovarian carcinomas and the results are collected in Table 2. All the compounds inhibit cell proliferation in the sub μM range against both non-resistant and resistant P-glycoprotein overexpressing, multidrug ovarian carcinoma cell lines, and no significant differences between both cell lines (R/S ratio close to 1) were found. Thus, P-glycoprotein overexpression does not seem to represent a problem for this chemotype.

**Table 2:** Anti-proliferative activity of the benzofurane derivatives in A2780 and A2780 AD cell lines.

Comp.	A2780 IC_50_ (μM)^a^	A2780 AD IC_50_ (µM)^a^	R/S index^b^
**8**	0.116 ± 0.008	0.111 ± 0.016	0.96
**20**	0.44 ± 0.02	0.66 ± 0.05	1.49
**27**	0.023 ± 0.002	0.039 ± 0.015	1.70
**28**	0.038 ± 0.007	0.076 ± 0.015	2.00
Podophilotoxin	0.064 ± 0.009	0.074 ± 0.012	1.15
Colchicine	0.015 ± 0.004	0.9 ± 0.072	58.9
Paclitaxel	0.001 ± 0.0001	2.1 ± 0.3	2100

^a^IC_50_ (50% inhibition of cell proliferation) values in ovarian carcinomas. IC_50_ values are given as the mean ± standard error of three independent experiments.

^b^Resistance index (the relative resistance of A2780AD cell line, obtained by dividing the IC_50_ of the resistant cell line by that of the parental A2780 cell line).

### Tubulin binding

Besides the antiproliferative activity, it was crucial to determine the capacity of the new compounds to bind tubulin. Binding affinities for the colchicine site in tubulin were determined by competition experiments with (R)-(+)-ethyl 5-amino 2-methyl-1,2-dihydro-3-phenylpyrido[3,4-*b*]pyrazin-7-yl carbamate (R-PT), as in previous examples^31,46^. However, under these conditions, compound **27** provided a strong association with tubulin that prevented the determination of the binding constant by displacement of R-PT^47^. Therefore a new reference ligand with a known higher binding affinity had to be used. Compound **22** with a Kb = 9.1 × 10^7^ M^−1^ was selected as the reference compound for the competition assay with **27** instead of R-PT (data collected in Table 1).

Given the similar spectroscopic properties of **22** and **27**, the competition experiment for determination of the binding constant of **27** was based on a centrifugation assay. Both compound **27** and the reference compound **22** were incubated in molar excess with respect to tubulin to achieve a saturated equilibrium state. By centrifugation tubulin co-sediments with the bound ligands were discharged and the concentration of the free compounds in solution could be determined by reverse-phase HPLC. Using equations based on the law of mass action (see Methods), the binding affinity of compound **27** was calculated (Kb = 287 ± 106 × 10^6^ M^−1^).

### Molecular modelling studies

**Figure 5:**
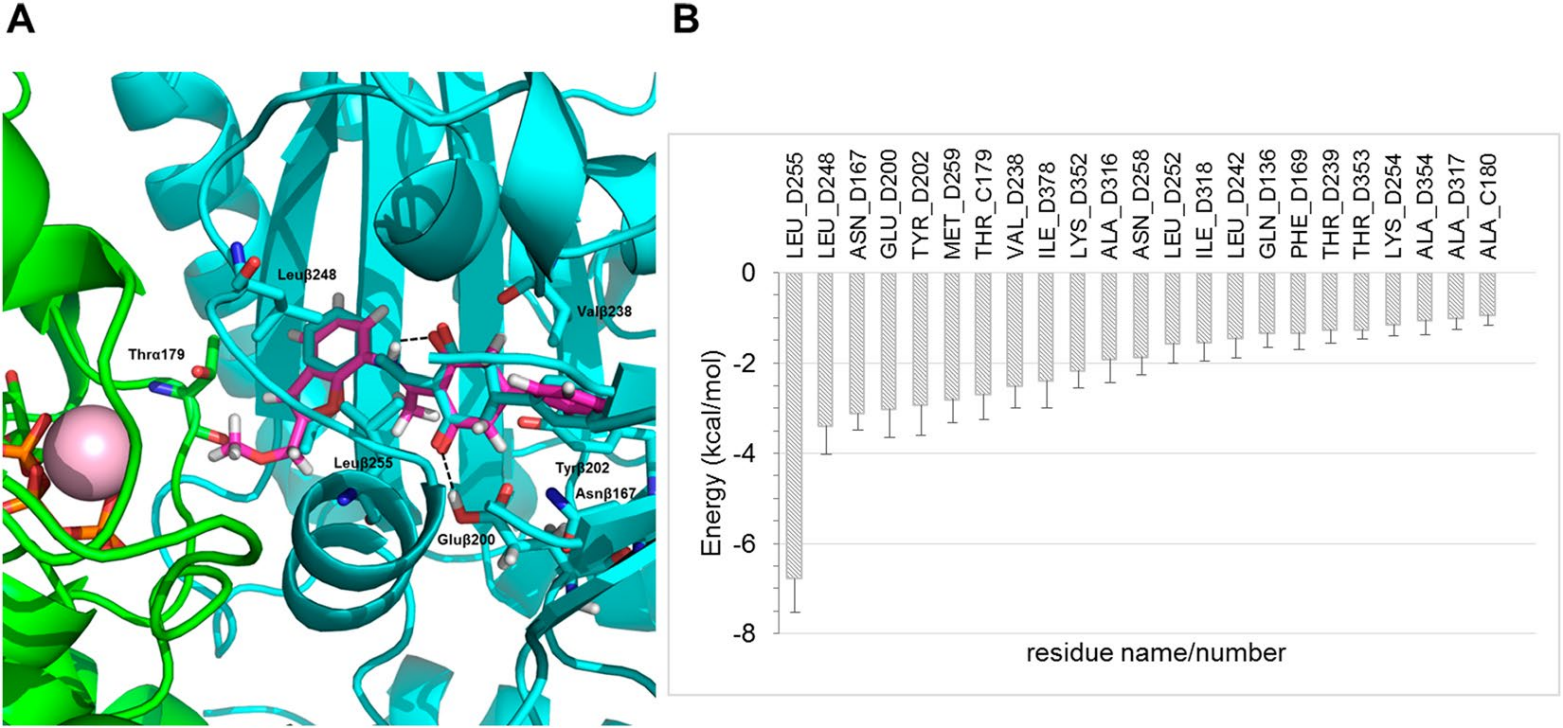
Docking and MD of compound 27 at TUB075 binding site. (**A**) Superposition of docked **27** onto the experimental solution found for TUB075. Note that the added extension points toward the α,β interface. α-Tubulin in green, β-tubulin in cyan, TUB075 in cyan sticks and **27** in magenta sticks. Important residues for the binding are shown in sticks and labelled and hydrogen bonds are represented as black dashed lines. (**B**) Solvent-corrected binding energies (kcal·mol^−1^)^48^ between **27** and individual residues in α- (**C**) and β-tubulin (**D**) collected over 500 snapshots taken from the MD simulation. For clarity, a cutoff of 0.9 kcal mol^−1^ was used.

To gain insight into the binding determinants conferring on compound **27** high affinity for tubulin, molecular modelling studies were performed using the coordinates of an α_2_β_2_ tubulin dimer extracted from the TUB075-(α_1_β_1_:α_2_β_2_) tubulin complex solved by x-ray crystallography. The complex obtained with the best docking pose was then relaxed by means of a molecular dynamics (MD) simulation lasting 50 ns, during which a soft harmonic restraint (5.0 kcal·mol^−1^Å^−2^) on the protein Cα atoms was used (Fig. 5A), followed by energy minimization. The resulting complex showed that the central core of **27** (regions A, B and C, Fig. 2A) shares the same pocket and orientation as TUB075, thus keeping hydrogen bonding interactions with Glu200 and Val238. Moreover, the 2-methylmethoxy group of **27**, which lies at the interface between the α and β subunits, is found pointing towards the T5 loop of α-tubulin.

A detailed study of the contribution of individual protein residues to the free energy of binding along the MD trajectory (which was generated to improve the sampling of the ligand around the crystallographically determined positions of the protein) was carried out by means of program MM-ISMSA^48^. This approach takes into account not only van der Waals and electrostatic interactions but also ligand and receptor desolvation. As shown in Fig. 5B, the most important residue for the binding of **27** is Leu255, followed, in decreasing order of importance, by Leu248, Asn167 and Glu200.

### Inhibition of cell cycle progression

**Figure 6:**
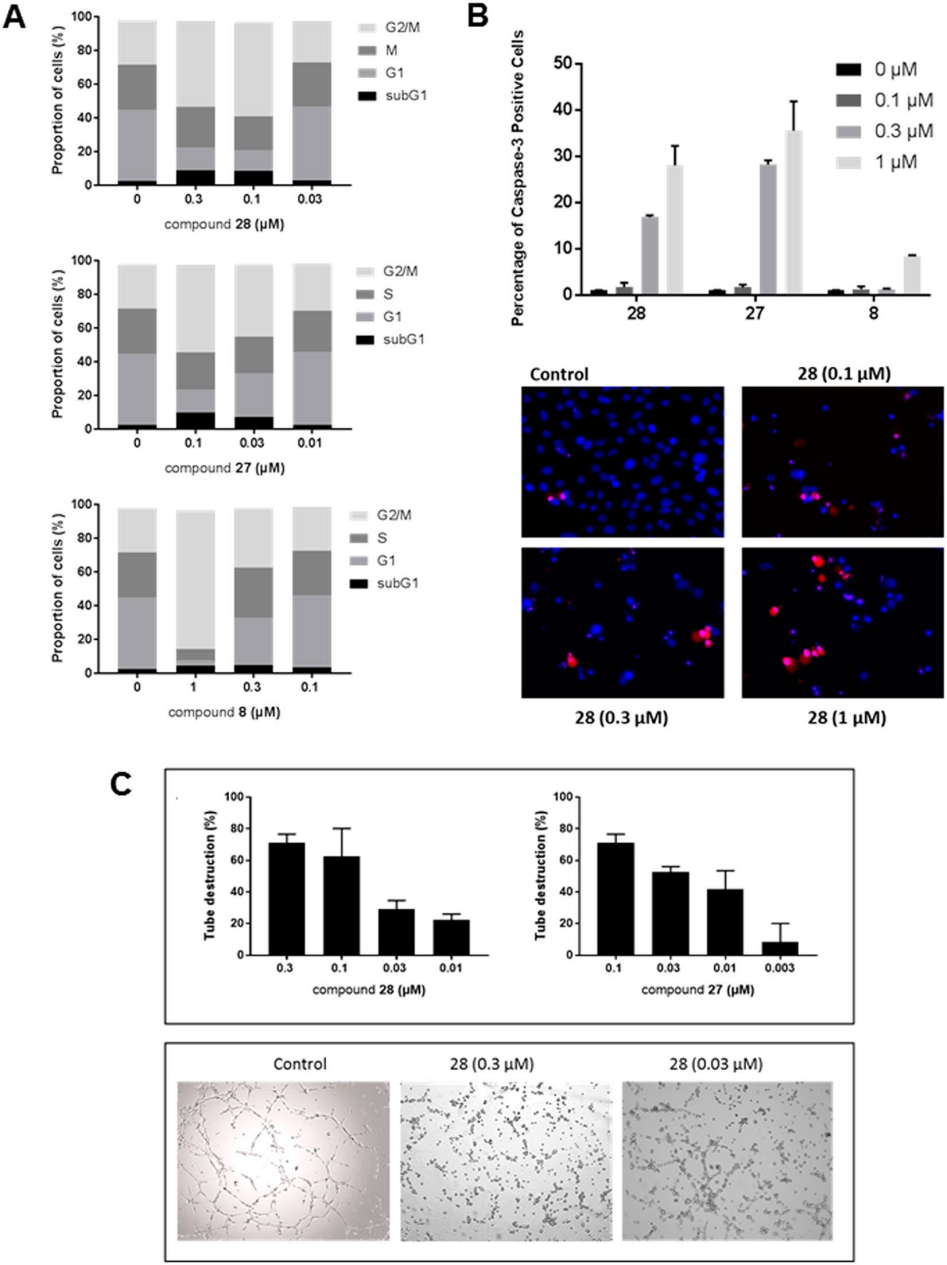
Mechanism of action of the benzofurane derivatives. (**A**) *Inhibition of cell cycle progression*. MDA-MB-231 cells were treated with DMSO (control) or compounds **8**, **27** or **28** for 24 h. Next, the cells were harvested, stained with propidium iodide (PI), and cell cycle distribution was evaluated by flow cytometry. Percentages of cells in the different phases of the cell cycle are indicated. (**B**) *Caspase-3 activity*. MDA-MB-231 cells were seeded in 48-well plates at 40,000 cells/cm². After 24 h, different concentrations of compounds **8**, **27** or **28** and 2 µM of the caspase-3 substrate DEVD-NucView488 were added. After 16 h, the cells were incubated with 2 µg/ml Hoechst 33342 to stain the nucleus, and imaged. (**C**) *Disruption of the vascular network*. HMEC-1 cells were cultured on matrigel for 3 h to allow the formation of tube-like structures. Next, different concentrations of compounds were added. Upper panel: After 90 min, pictures were taken and the tubular network was scored (3: intact network, 0: network completely destroyed). Bars show average ± SEM (n = 4). Lower panel: Microscopic pictures of the tubular network 3 h after addition of compound.

Tubulin-binding agents interfere with mitotic spindle formation during cell division. This results in the inhibition of cell proliferation and/or induction of apoptosis. Thus, we investigated whether **27** and **28** affect the progression of human breast cancer MDA-MB-231 cells through the cell cycle. Control cells displayed a characteristic distribution of cells in different phases of the cell cycle (Fig. 6A). In contrast, compounds **27** and **28** arrested the cell cycle in the M phase (mitosis) at concentrations as low as 0.03 and 0.1 μM (for compound **27** and **28** respectively). In contrast, the hit compound **8** proved no longer active at 0.3 µM). Moreover, compounds **27** and **28** caused an increase of cells in the sub-G1 phase, which is indicative of apoptosis.

To confirm the capacity of compounds **27** and **28** to induce apoptosis, MDA-MB-231 cells were treated with the compounds for 24 h in the presence of NucView^®^ 530 Caspase-3. This substrate is cleaved by active caspase-3 activity (the main executioner enzyme during apoptosis) to generate a fluorescent derivative that is analyzed using fluorescence microscopy. As can be seen in Fig. 6B both compounds were capable of inducing apoptosis in a dose-dependent manner, compound **27** being more active than compound **28**. After 24 h, the maximum apoptotic rate of ~30 and 40% was achieved at 1 µM of compound **27** or **28**, respectively. At this concentration, only 9% of apoptotic cells were observed in cultures treated with compound **8**.

### Vascular disrupting activity

Another feature of colchicine-site binding agents is their ability to destroy a 3-dimensional network of endothelial cells. To determine the vascular disrupting capacity of compounds **27** and **28**, HMEC-1 cells were seeded on top of matrigel. This tumor derived extracellular matrix induces within 3 h the rearrangement of endothelial cells into a vascular network. Compounds **27** and **28** caused a disruption of the tubular network in a concentration-dependent manner (Fig. 6C). At concentrations of 0.1 μM, both compounds nearly completely abrogated the vascular network. Also, at a concentration of 0.01 μM, the antivascular effect was still evident, particularly for compound **27**.

### Inhibition of endothelial cell migration

**Figure 7:**
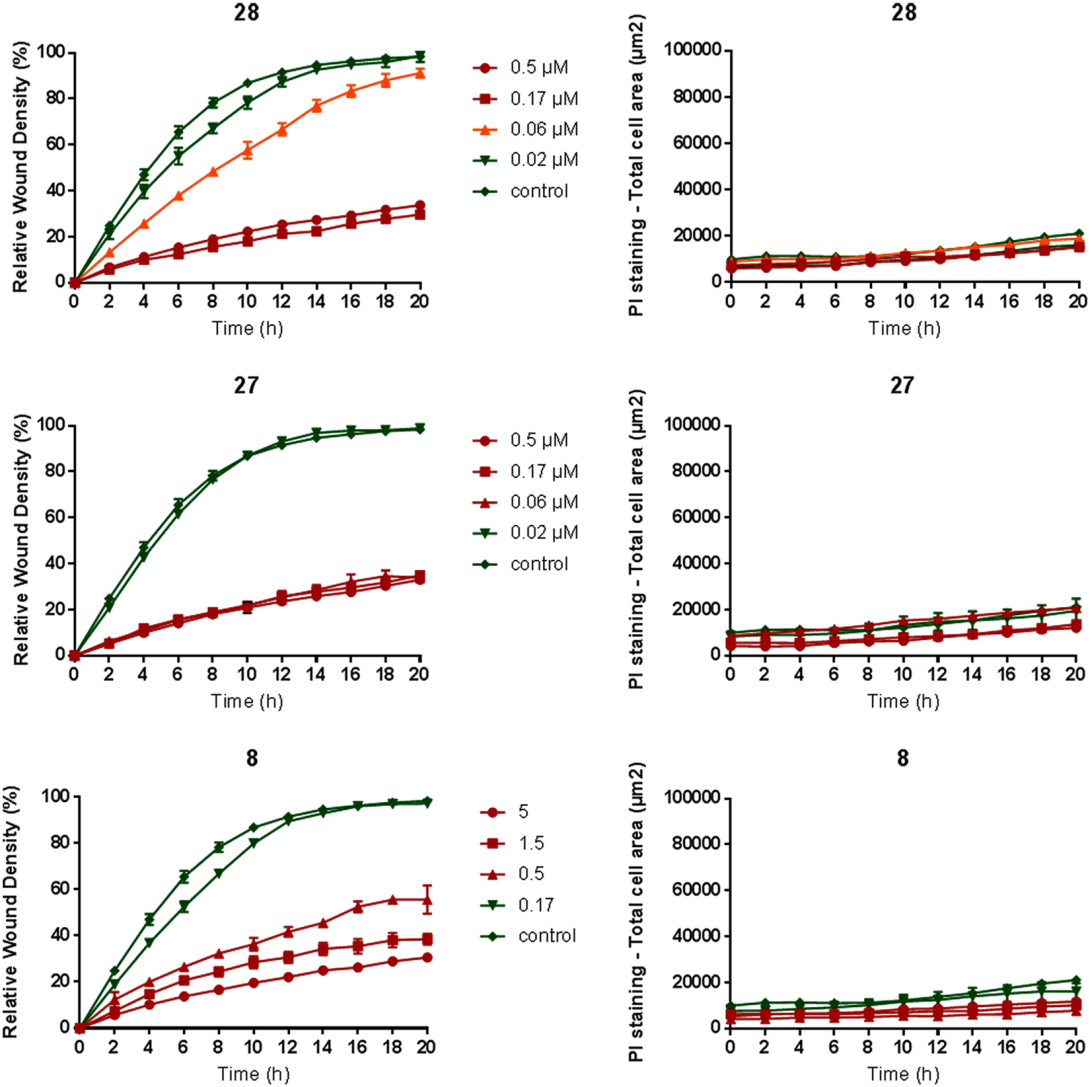
Inhibition of HMEC-1 migration. The 96-well IncuCyte® scratch wound assay was used to create a cell-free zone (wound) in a confluent cell monolayer. Next, compounds were added and the relative wound density was measured every minute and visualized in time-course plots (left panels). PI is added to all wells to visualize toxicity over time, i.e. only dead cells take up this cell-impermeable dye (right panels). Average ± SEM (n = 3) are shown.

Proper tubulin organization is not only required for cell division but also for cell motility and migration. Therefore, we evaluated the capacity of the compounds to inhibit the migration of HMEC-1 endothelial cells. To this end, we used the 96-well IncuCyte^®^ scratch wound assay. After the creation of a cell-free zone (wound) in a confluent cell monolayer, compounds were added, and relative wound density was measured every minute and visualized in time-course plots. PI was added to all wells to visualize toxicity over time, i.e. only dead cells take up this cell-impermeable dye. As shown in Fig. 7, compounds **8**, **27** and **28** inhibited wound closure at concentrations as low as 0.5, 0.06 and 0.06 µM, respectively. No increased uptake of PI was noted at these concentrations, indicating a specific, non-toxic effect.

## Conclusions

We have solved the structures of tubulin in complex with two cyclohexanedione derivatives TUB015 and TUB075 by X-ray crystallography at 2.15 and 2.1 Å resolution. Most interactions between the ligand and the protein are hydrophobic in nature, as expected, although there are also some polar interactions, particularly between the C-3 carbonyl of the TUB ligands and the side chain of βGlu200 and a second water mediated hydrogen bond between the C1-carbonyl and the main chain amide of βCys241. Little overlap is observed among the tubulin-bound TUB and the colchicine molecules since TUB075 and TUB015 occupied zones 2 and 3 of the colchicine domain, as defined by Massarotti *et al*.^36^, while colchicine makes use of the most external zone, designated zone 1. Analysis of the binding site of TUB075 with the cGRILL program allowed the identification of two additional regions around fragment D of TUB075 that could be exploited to gain additional binding affinity. To occupy these two regions, a new series of cyclohexanediones with a 2-substituted benzofurane ring as fragment D were envisaged. Interestingly, from the new series, compound **27** with a 2-methoxymethyl group and compound **28** with a 2-(3-hydroxypropoxy)methyl substituent at the benzofurane, showed antiproliferative activity against tumor and endothelial cells in the nanomolar range (IC_50_ = 8–31 nM), keeping full activity against P-glycoprotein overexpressing cells (A2780 AD). Additional studies performed to determine the mechanism of action of **27** and **28** revealed that they caused cell cycle arrest in G_2_/M at doses lower than those of the previous hit **8** (TUB075). In addition, they were able to induce apoptosis as shown in the caspase-3 assays. Compounds **27** and **28** further destroyed a preformed vascular network and inhibited the migration of endothelial cells at non-toxic concentrations. Very notably, compounds **27** and **28** demonstrated an exceptionally high affinity for tubulin in the binding assays, so that the K_b_ of compound **27** (2.87 × 10^8^ M^−1^), represents, to the best of our knowledge, the highest affinity constant described in the literature for a colchicine-site ligand.

## Methods

### Chemistry procedures

Melting points were obtained on a Reichert-Jung Kofler apparatus and are uncorrected. The elemental analysis was performed with a Heraeus CHN-O-RAPID instrument. The elemental compositions of the compounds agreed to within ± 0.4% of the calculated values.

Electrospray mass spectra were measured on a quadrupole mass spectrometer equipped with an electrospray source (Hewlett-Packard, LC/MS HP 1100).

^1^H and ^13^C NMR spectra were recorded on a Varian INNOVA 300 operating at 299 MHz (^1^H) and 75 MHz (^13^C), respectively, a Varian INNOVA-400 operating at 399 MHZ (^1^H) and 99 MHz (^13^C), respectively, and a VARIAN SYSTEM-500 operating a 499 MHz (^1^H) and 125 MHz (^13^C), respectively. Monodimensional ^1^H and ^13^C spectra were obtained using standard conditions. 2D inverse proton detected heteronuclear one-bond shift correlation spectra were obtained using the Pulsed Field Gradient HSQC pulse sequence. Data were collected in a 2048 × 512 matrix with a spectral width of 3460 Hz in the proton domain and 22500 Hz in the carbon domain, and processed in a 2048 × 1024 matrix. The experiment was optimized for one bond heteronuclear coupling constant of 150 Hz. 2D inverse proton detected heteronuclear long-range shift correlation spectra were obtained using the Pulsed Field Gradient HMBC pulse sequence. The HMBC experiment was acquired in the same conditions that HSQC experiment and optimized for long range coupling constants of 7 Hz.

Analytical TLC was performed on silica gel 60 F_254_ (Merck) precoated plates (0.2 mm). Spots were detected under UV light (254 nm) and/or charring with ninhydrin or phosphomolibdic acid.

Separations on silica gel were performed by preparative centrifugal circular thin-layer chromatography (CCTLC) on a Chromatotron^R^ (Kiesegel 60 PF_254_ gipshaltig (Merck)), with layer thickness of 1 and 2 mm and flow rate of 4 or 8 mL/min, respectively. Flash column chromatography was performed in a Biotage Horizon instrument.

Microwave reactions were performed using the Biotage Initiator 2.0 single-mode cavity instrument from Biotage (Uppsala). Experiments were carried out in sealed microwave process vials utilizing the standard absorbance level (400 W maximum power). The temperature was measured with an IR sensor on the outside of the reaction vessel.

#### General procedure for the reaction of 2-acetylcyclohexane-1,3-dione with anilines (General procedure A)

A solution of 2-acetylcyclohexane-1,3-dione (**13**) (1.0 mmol) and the appropriate aniline (1.5 mmol) in toluene (10 mL) was placed in an Ace pressure tube^31^. Then, 4 Å molecular sieves were added, the vessel was sealed and heated at 110 °C overnight. After cooling, the solvent was evaporated and the residue was purified by flash chromatography or by CCTLC in the Chromatotron.

#### General procedure for the synthesis of 7-aminobenzofuranes by reduction of 7-nitrobezofuranes anilines (General procedure B)

The corresponding 7-nitrobenzofurane (1 eq) was dissolved in ethyl acetate (12 mL) in a pressure vessel and then 5% Pt/S (catalytic amount) was added. The mixture was hydrogenated at 30 psi for 1–8 h at room temperature. Then, the reaction mixture was filtered and volatiles were removed under reduced pressure. The obtained 2-aminobenzofuranes were directly used as such in the subsequent reaction without further purification or purified as specified.

#### 2-Methyl-7-nitrobenzofuran (11)

Following a described procedure^39^, a solution of 1-nitro-2-(prop-2-yn-1-yloxy)benzene (**10**)^38^ (206 mg, 1.16 mmol) in polyethylene glycol-500 (1.2 mL) was heated at 215 °C for 90 min under nitrogen atmosphere. Then the reaction was cooled to room temperature, washed with water and extracted with ethyl acetate (3 × 15 mL). The organic layer was dried over Na_2_SO_4_, filtered and evaporated to dryness. The crude was purified by CCTLC in the Chromatothron (hexane/ethyl acetate, 10:1) to yield 80 mg (39%) of **11**^40^ as a yellow solid. Mp 98–100 °C (lit^40^ 101 °C). ^1^H NMR (CDCl_3,_ 300 MHz) δ: 2.58 (d, *J* = 1.1 Hz, 3 H, CH_3_), 6.53 (q, *J* = 1.1 Hz, 1 H, Ar), 7.29 (dd, *J* = 8.2, 7.7 Hz, 1 H, Ar), 7.77 (dd, *J* = 7.7, 1.2 Hz, 1 H, Ar), 8.05 (dd, *J* = 8.2, 1.2 Hz, 1 H, Ar). ^1^H NMR data are similar to those previously described^40^.

#### 2-Methylbenzofuran-7-amine (12)

To a solution of **11** (64 mg, 0.36 mmol) in methanol (1.4 mL) at rt, SnCl_2_ (205 mg, 1.08 mmol) and 37% HCl (0.3 mL) were added. The reaction mixture was heated at 75 °C for 30 min. Methanol was removed under reduced pressure. The reaction mixture was basified (up to pH 10) by adding a solution of 10% NaOH (4 mL) at 0–5 °C, and extracted with ethyl acetate (3 × 15 mL). The organic layer was dried over Na_2_SO_4_, filtered and evaporated to dryness. The crude was purified by CCTLC in the Chromatothron (hexane/ethyl acetate, 2:1) to yield 44 mg (83%) of **12**^41^ as a yellow oil. MS (ES, positive mode): 148 (M + H)^+ 1^H NMR (CDCl_3,_ 300 MHz) δ: 2.41 (d, *J* = 1.1 Hz, 3 H, CH_3_), 5.22 (br s, 2 H, NH_2_), 6.42 (q, *J* = 1.1 Hz, 1 H, Ar), 6.47 (dd, *J* = 7.7, 1.2 Hz, 1 H, Ar), 6.69 (dd, *J* = 7.6, 1.1 Hz, 1 H, Ar), 6.85 (t, *J* = 7.7 Hz, 1 H, Ar). ^1^H NMR data are similar to those previously described^41^.

#### 2-(1-((2-Methylbenzofuran-7-yl)amino)ethylidene)-5-phenylcyclohexane-1,3-dione (14)

Following the general procedure A, an Ace pressure tube was charged with **13** (52 mg, 0.22 mmol), **12** (22 mg, 0.15 mmol) and 4 Å molecular sieves in toluene (0.6 mL) overnight. The residue was worked up and purified by CCTLC in the Chromatothron (hexane/ethyl acetate, 4:1) to yield 40 mg (74%) of **14** as a white solid. Mp 127–129 °C. MS (ES, positive mode): m/z 360 (M + H)^+ 1^H NMR (DMSO-*d*_6,_ 400 MHz) δ: 2.46 (s, 3 H, CH_3_), 2.47 (s, 3 H, CH_3_), 2.61–2.72 (m, 2 H, H-4, H-6), 3.75–3.02 (m, 2 H, H-4, H-6), 3.39 (m, 1 H, H-5), 6.70 (m, 1 H, Ar,), 7.19–7.39 (m, 7 H, Ar), 7.57 (dd, *J* = 7.6, 1.3 Hz, 1 H, Ar), 15.14 (br s, 1 H, NH). ^13^C NMR (DMSO-*d*_6,_ 100 MHz) δ: 13.8 (CH_3_), 19.9 (CH_3_), 36.0 (C-5), 46.1 (C-4, C-6), 103.4 (Ar), 108.8 (NHC = C), 119.9, 120.1, 120.5, 123.3, 126.6, 126.8, 128.5, 130.5, 143.5, 147.7, 156.8 (Ar), 172.7 (NHC = C). Anal. calc. for (C_23_H_21_NO_3_): C, 76.86; H, 5.89; N, 3.90. Found: C, 76.56; H, 6.18; N, 4.09.

#### Methyl 7-aminobenzofuran-2-carboxylate (16)

Following the general procedure B, a solution of methyl 7-nitrobenzofuran-2-carboxylate (**15**)^42^ (56 mg, 0.25 mmol) in ethyl acetate (12 mL) with 5% Pt/S (catalytic amount) was subjected to hydrogenation (30 psi) for 5 h at room temperature. Then, the reaction mixture was filtered and the solvent was evaporated to yield 48 mg (99%) of **16**^43^ as yellow oil. MS (ES, positive mode): m/z 192 (M + H)^+^. ^1^H NMR (DMSO-d_6_, 300 MHz) δ: 3.88 (s, 3 H, CH_3_), 5.51 (br s, 2 H, NH_2_), 6.71 (dd, *J* = 7.6, 1.2 Hz, 1 H, Ar), 6.93 (dd, *J* = 7.7, 1.1 Hz, 1 H, Ar), 7.04 (t, *J* = 7.7 Hz, 1 H, Ar), 7.65 (s, 1 H, Ar). Although this compound was mentioned in literature^43^ no analytical data were provided.

#### 7-Amino-N-methylbenzofuran-2-carboxamide (17)

A microwave vial was charged with a mixture of **16** (82 mg, 0.43 mmol), 2 M *N*-methylamine in methanol (3.6 mL, 7.27 mmol), NH_4_Cl (8 mg, 0.15 mmol) in methanol (4 mL). The reaction vessel was sealed and heated in a microwave reactor at 60 °C for 3 h. After cooling, volatiles were removed. The residue was washed with water and extracted with DCM (3 × 15 mL). The organic layer was dried over Na_2_SO_4_, filtered and evaporated to dryness to yield 82 mg of **17** (100%) as a yellow oil. MS (ES, positive mode): m/z 191 (M + H)^+^, 381 (2 M + H)^+^. ^1^H NMR (DMSO-*d*_6,_ 400 MHz) δ: 2.83 (d, *J* = 4.7 Hz, 3 H, CH_3_), 5.34 (br s, 2 H, NH_2_), 6.65 (d, *J* = 7.6 Hz, 1 H, Ar), 6.88 (d, *J* = 7.7 Hz, 1 H, Ar), 6.99 (t, *J* = 7.7 Hz, 1 H, Ar), 7.34 (s, 1 H, Ar), 8.47 (m, 1 H, NH).

#### (7-Nitrobenzofuran-2-yl)metanol (18)

To a solution of **15** (191 mg, 0.86 mmol) in diethyl ether (3 mL) at −78 °C (liquid nitrogen/ethyl acetate bath), 1 M DIBALH in toluene (860 µL. 0.86 mmol) was added. The mixture was stirred and allowed to reach room temperature over 1 h. The mixture was again cooled to −78 °C (liquid nitrogen/ethyl acetate bath) and further portions of 1 M DIBALH in toluene (860 µL, 0.86 mmol) were added. The mixture was stirred and allowed to reach ambient temperature over 4 h. The mixture was cooled to 0 °C (ice/water bath) and quenched by adding de-ionized water and 1 M NaOH. Then it was extracted with DCM (2 × 15 mL). The combined organic layers were dried and evaporated to afford 160 mg of **18** (96%) as a yellow solid. Mp 62–64 °C. ^1^H NMR (DMSO-*d*_6,_ 400 MHz) δ: 4.66 (dd, *J* = 5.8, 0.9 Hz, 2 H, CH_2_), 5.68 (t, *J* = 5.9 Hz, 1 H, OH), 7.02 (s, 1 H, Ar), 7.46 (dd, *J* = 7.7 Hz, 1 H, Ar), 8.08 (dd, *J* = 7.7, 1.1 Hz, 1 H, Ar), 8.13 (dd, *J* = 8.2, 1.1 Hz, 1 H, Ar).

#### (7-Aminobenzofuran-2-yl)methanol (19)

Following the general procedure B, a solution of **18** (45 mg, 0.22 mmol) in ethyl acetate (12 mL) in the presence of 5% Pt/S (catalytic amount) was subjected to hydrogenation (30 psi) for 4 h at room temperature. Then, the reaction mixture was filtered and the solvent was evaporated to yield 29 mg (84%) of **19** as yellow oil. MS (ES, positive mode): m/z 164 (M + H). ^1^H NMR (400 MHz, DMSO-*d*_6_) δ: 4.54 (d, *J* = 5.7 Hz, 2 H, CH_2_), 5.17 (br s, 2 H, NH_2_), 5.37 (t, *J* = 5.7 Hz, 1 H, OH), 6.51 (dd, *J* = 7.7, 1.2 Hz, 1 H, Ar), 6.61 (s, 1 H, Ar), 6.75 (dd, *J* = 7.7, 1.2 Hz, 1 H, Ar), 6.89 (t, *J* = 7.7 Hz, 1 H, Ar).

#### Methyl 7-((1-(2,6-dioxo-4-phenylcyclohexylidene)ethyl)amino)benzofuran-2-carboxylate (20)

Following the general procedure A, an Ace pressure tube was charged with **13** (37 mg, 0.16 mmol), **16** (46 mg, 0. 24 mmol) and 4 Å molecular sieves in toluene (0.7 mL). The residue was worked up and purified by CCTLC in the Chromatothron (hexane/ethyl acetate, 2:1) to yield 28 mg (43%) of **20** as a white solid. Mp 178–180 °C. MS (ES, positive mode): m/z 404 (M + H)^+^. ^1^H NMR (DMSO-*d*_6,_ 400 MHz) δ: 2.46 (s, 3 H, CH_3_), 2.25–2.76 (m, 2 H, H-4, H-6), 2.77–3.04 (m, 2 H, H-4, H-6), 3.41 (tt, *J* = 12.2, 4.0 Hz, 1 H, H-5), 3.90 (s, 3 H, OCH_3_), 7.25 (m, 1 H, Ar), 7.33–7.38 (m, 4 H, Ar), 7.47 (t, *J* = 7.8 Hz, 1 H, Ar), 7.56 (m, 1 H, Ar), 7.85 (m, 1 H, Ar), 7.90 (s, 1 H, Ar), 15.18 (br s, 1 H, NH). ^13^C NMR (DMSO- *d*_6,_ 100 MHz) δ: 19.9 (CH_3_), 35.9 (C-5), 46.5 (C-4, C-6), 52.6 (OCH_3_), 109.0 (NHC = C), 114.7, 121.4, 123.0, 124.7, 125.2, 126.6, 126.78, 128.3, 128.56, 143.4, 145.7, 148.9 (Ar), 158.80 (COOCH_3_), 172.8 (NHC = C). Analysis (calcd., found for C_24_H_21_NO_5_): C (71.45, 71.06), H (5.25, 5.52), N (3.47, 3.71).

#### 7-((1-(2,6-Dioxo-4-phenylcyclohexylidene)ethyl)amino)-N-methylbenzofuran-2-carboxamide (21)

Following the general procedure A, an Ace pressure tube was charged with **13** (66 mg, 0.28 mmol), **17** (82 mg, 0.43 mmol) and 4 Å molecular sieves in toluene (1.1 mL). The residue was worked up and purified by CCTLC in the Chromatothron (dichloromethane/methanol, 10:1) and (hexane/ethyl acetate, 1:2) to yield 86 mg (76%) of **20** as a white solid. Mp 108–110 °C. MS (ES,positive mode): m/z 403 (M + H)^+^. ^1^H NMR (DMSO-*d*_6,_ 400 MHz) δ: 2.46 (s, 3 H, CH_3_), 2.56–2.74 (m, 2 H, H-4, H-6), 2.80 (d, *J* = 4.6 Hz, 3 H, HNCH_3_), 2.85–3.02 (m, 2 H, H-4, H-6), 3.39 (tt, *J* = 12.5, 4.2 Hz, 1 H, H-5), 7.25 (m, 1 H, Ar), 7.32–7.39 (m, 4 H, Ar), 7.39–7.47 (m, 2 H, Ar), 7.62 (s, 1 H, Ar), 7.81 (dd, *J* = 7.1, 1.9 Hz, 1 H, Ar), 8.69 (q, *J* = 4.6 Hz, 1 H, HNCH_3_), 15.14 (br s, 1 H, NH). ^13^C NMR (DMSO-*d*_6,_ 100 MHz) δ: 19.8 (CH_3_), 25.8 (HNCH_3_), 36.0 (C-5), 45.3 (C-4, C-6), 108.8 (NHC = C), 109.4, 121.1, 122.6, 124.2, 124.4, 126.6, 126.8, 128.6, 128.9, 143.4, 148.2, 150.0 (Ar), 158.2 (NHCO), 173.1 (NHC = C). Analysis (calcd., found for C_24_H_22_N_2_O_4_·0.5H_2_O): C (70.06, 69.82), H (5.63, 5.82), N (6.81, 6.98).

#### 2-(1-((2-(Hydroxymethyl)benzofuran-7-yl)amino)ethylidene)-5-phenylcyclohexane-1,3-dione (22)

Following the general procedure A, an Ace pressure tube was charged with **13** (52 mg, 0.23 mmol), **19** (26 mg, 0.16 mmol) and 4 Å molecular sieves in toluene (0.7 mL). The residue was worked up and purified by CCTLC in the Chromatothron (dichloromethane/methanol, 20:1) to yield 57 mg (79%) of **22** as a white solid. Mp 120–122 °C. MS (ES, positive mode): m/z 376 (M + H)^+^. ^1^H NMR (DMSO-*d*_6,_ 400 MHz) δ: 2.46 (s, 1 H, CH_3_), 2.60–2.73 (m, 2 H, H-4, H-6), 2.75–2.97 (m, 2 H, H-4, H-6), 3.39 (tt, *J* = 12.3, 4.1 Hz, 1 H, H-5), 4.58 (d, *J* = 5.9 Hz, 2 H, CH_2_OH), 5.55 (t, *J* = 5.8 Hz, 1 H, OH), 6.89 (s, 1 H, Ar), 7.21–7.37 (m, 7 H, Ar), 7.64 (dd, *J* = 7.3, 1.6 Hz, 1 H, Ar), 15.15 (br s, 1 H, NH). ^13^C NMR (DMSO-*d*_6,_ 101 MHz) δ: 19.8 (CH_3_), 36.0 (C-5), 45.4 (C-4, C-6), 56.0 (CH_2_OH), 103.9 (Ar), 108.8 (NH-C = C), 120.3, 120.9, 121.3, 123.4, 126.6, 126.8, 128.5, 129.8, 143.4, 148.0, 159.7 (Ar), 172.8 (NH-C = C). Analysis (calcd., found for C_23_H_21_NO_4_·0.5H_2_O): C (71.86, 71.84), H (5.77, 5.98), N (3.64, 3.63).

#### 2-(Methoxymethyl)-7-nitrobenzofuran (23)

To a solution of **18** (30 mg, 0.16 mmol) in anhydrous DMF at 0 °C, 60% NaH (13 mg, 0.32 mmol) was added. After 10 min methyl iodide (30 µL, 0.46 mmol) was added. The mixture was stirred at room temperature for 2 h, quenched by adding a saturated aqueous NH_4_Cl solution and extracted with ethyl acetate (3 × 15 mL). The organic layer was dried over Na_2_SO_4_, filtered and evaporated to dryness to yield 32 mg (99%) of **23** as a yellow oil. ^1^H NMR (DMSO-*d*_6,_ 400 MHz) δ: 3.35 (s, 3 H, CH_3_), 4.63 (s, 2 H, CH_2_), 7.17 (s, 1 H, Ar), 7.48 (t, *J* = 7.9 Hz, 1 H, Ar), 8.11 (dd, *J* = 7.7, 1.2 Hz, 1 H, Ar), 8.16 (dd, *J* = 8.1, 1.2 Hz, 1 H, Ar).

#### 1-*O*-*tert*-Butyldimethylsilyl-3-((7-nitrobenzofuran-2-yl)methoxy)propan-1-ol (24)

To a solution of **23** (67 mg, 0.35 mmol) in THF (1.4 mL), a 50% NaOH solution (208 μL) was added dropwise at room temperature followed by the addition of tetrabutylammonium bromide (56 mg, 0.17 mmol). The mixture was heated to 60 °C and (3-bromopropoxy)(*tert*-butyl)dimethylsilane^44^ (132 mg, 0.52 mmol) was slowly added. The final mixture was stirred for 4 h at 60 °C. The residue was worked up and purified by CCTLC in the Chromatothron (hexane/ethyl acetate, 4:1) to yield 140 mg (85%) of **24** as a yellow oil. MS (ES, positive mode): m/z 366 (M + H)^+^. ^1^H NMR (DMSO-d_6,_ 400 MHz) δ: 0.78 (s, 9 H, C(CH_3_)_3_), 1.71 (p, *J* = 6.2 Hz, 2 H, OCH_2_CH_2_CH_2_), 3.58 (t, *J* *=* 6.3 Hz, 2 H, OCH_2_CH_2_CH_2_), 3.63 (t, *J* = 6.1 Hz, 2 H, OCH_2_CH_2_CH_2_), 4.67 (s, 2 H, OCH_2_Ar), 7.16 (s, 1 H, Ar), 7.48 (t, *J* = 7.9 Hz, 1 H, Ar), 8.11 (dd, *J* = 7.8, 1.1 Hz, 1 H, Ar), 8.16 (dd, *J* = 8.1, 1.1 Hz, 1 H, Ar).

#### 2-(Methoxymethyl)benzofuran-7-amine (25)

Following the general procedure B, a solution of **23** (45 mg, 0.22 mmol) in ethyl acetate (12 mL) was subjected to hydrogenation for 8 h at room temperature. Then, the reaction mixture was filtered and the solvent was evaporated to yield 40 mg (84%) of **25** as a yellow oil. MS (ES, positive mode): m/z 178 (M + H). ^1^H NMR (DMSO-*d*_6,_ 400 MHz) δ: 3.30 (s, 3 H, OCH_3_), 4.49 (s, 2 H, CH_2_), 5.25 (br s, 2 H, NH_2_), 6.54 (dd, *J* = 7.7, 1.2 Hz, 1 H, Ar), 6.74–6.81 (m, 2 H, Ar), 6.91 (t, *J* = 7.7 Hz, 1 H, Ar), 7.32–7.37 (m, 1 H, Ar).

#### 2-((3-((*tert*-Butyldimethylsilyl)oxy)propoxy)methyl)benzofuran-7-amine (26)

Following the general procedure B, a solution of **24** (100 mg, 0.27 mmol) in ethyl acetate (12 mL) was subjected to hydrogenation for 2 h at room temperature. Then, the reaction mixture was filtered and the solvent was evaporated to yield 65 mg (71%) of **26** as a yellow oil. MS (ES, positive mode): m/z 336 (M + H)^+^. ^1^H NMR (DMSO-d_6,_ 400 MHz) δ: 0.84 (s, 9 H, C(CH_3_)_3_), 1.70 (p, *J* = 6.3 Hz, 2 H, OCH_2_CH_2_CH_2_OSi), 3.53 (t, *J* = 6.3 Hz, 2 H, OCH_2_CH_2_CH_2_OSi), 3.64 (t, *J* = 6.2 Hz, 2 H, OCH_2_CH_2_CH_2_OSi), 4.53 (s, 2 H, OCH_2_Ar), 5.22 (br s, 2 H, NH_2_), 6.53 (dd, *J* = 7.7, 1.1 Hz, 1 H, Ar), 6.76 (dd, *J* = 7.7, 1.1 Hz, 1 H, Ar), 6.73 (s, 1 H, Ar), 6.90 (t, *J* = 7.7 Hz, 1 H, Ar).

#### 2-(1-((2-(Methoxymethyl)benzofuran-7-yl)amino)ethylidene)-5-phenylcyclohexane-1,3-dione (27)

Following the general procedure A, an Ace pressure tube charged with **13** (58 mg, 0.25 mmol), **25** (30 mg, 0.17 mmol) and 4 Å molecular sieves in toluene (0.7 mL). The residue was worked up and purified by CCTLC in the Chromatothron (hexane/ethyl acetate, 1:1) and (dichloromethane/ methanol, 20:1) to yield 54 mg (82%) of **27** as a white solid. Mp 78–80 °C. MS (ES, positive mode): m/z 390 (M + H)^+ 1^H NMR (DMSO-*d*_6,_ 400 MHz) δ: 2.46 (s, 3 H, CH_3_), 2.60–2.73 (m, 2 H, H-4, H-6), 3.75–3.01 (m, 2 H, H-4, H-6), 3.31 (s, 3 H, OCH_3_), 3.40 (m, 1 H, H-5), 4.56 (s, 2 H, OCH_2_), 7.04 (s, 1 H, Ar), 7.24 (m, 1 H, Ar), 7.32–7.37 (m, 6 H, Ar), 7.68 (m, 1 H, Ar), 15.16 (br s, 1 H, NH). ^13^C NMR (DMSO-*d*_6,_ 100 MH,) δ: 19.8 (CH_3_), 36.0 (C-5), 46.2 (C-4, C-6), 57.5 (OCH_3_), 65.7 (OCH_2_), 106.6 (Ar), 108.8 (NH-C = C), 120.5, 121.1, 121.8, 123.6, 126.6, 126.8, 128.5, 129.4, 143.4, 148.2, 155.5 (Ar), 172.7 (NH-C = C). Analysis (calcd., found for C_24_H_23_NO_4_): C (74.02, 73.85), H (5.95, 6.20), N (3.60, 3.86).

#### 2-(1-((2-((3-Hydroxypropoxy)methyl)benzofuran-7-yl)amino)ethylidene)-5-phenylcyclohexane-1,3-dione (28)

Following the general procedure A, an Ace pressure tube was charged with **13** (45 mg, 0.13 mmol), **26** (21 mg, 0.09 mmol) and 4 Å molecular sieves in toluene (0.4 mL). The residue was worked up and purified by CCTLC in the Chromatothron (hexane ethyl acetate, 3:1) to yield 32 mg (59%) of the protected condensation product as a colorless oil [MS (ES, positive mode): m/z 548 (M + H)^+^. ^1^H NMR (DMSO-d_6,_ 400 MHz) δ: 0.79 (s, 9 H, C(CH_3_)_3_), 1.69 (p, *J* = 6.1 Hz, 2 H, OCH_2_CH_2_CH_2_OSi), 2.46 (s, 1 H, CH_3_), 2.59–2.74 (m, 2 H, H-4, H-6), 2.75–2.98 (m, 2 H, H-4, H-6), 3.39 (tt, *J* = 12.3, 4.1 Hz, 1 H, H-5), 3.53 (t, *J* = 6.3 Hz, 1 H, OCH_2_CH_2_CH_2_OSi), 3.61 (t, *J* = 6.1 Hz, 1 H, OCH_2_CH_2_CH_2_OSi), 4.59 (s, 2 H, OCH_2_Ar), 7.02 (s, 1 H, Ar), 7.25 (m, 1 H, Ar), 7.31–7.37 (m, 6 H, Ar), 7.66 (m, 1 H, Ar), 15.17 (br s, 1 H, NH).] Then, a solution of this product (24 mg, 0.04 mmol) in dichloromethane (0.3 mL) was treated with TFA (0.3 mL) and stirred at rt for 1 h. Volatiles were removed to afford a residue that was purified by CCTLC in the Chromatothron (dichloromethane/methanol, 10:0.2) to yield 16 mg (84%) of **28** as a white solid. Global yield for the two steps: 65%. Mp 73–75 °C. MS (ES, positive mode): m/z 434 (M + H)^+^.^1^H NMR (DMSO-d_6,_ 500 MHz) δ: 1.67 (p, *J* = 6.5 Hz, 2 H, OCH_2_CH_2_CH_2_OH), 2.46 (s, 3 H,CH_3_), 2.60–2.72 (m, 2 H, H-4, H-6), 2.76–2.98 (m, 2 H, H-4, H-6), 3.38 (m, 1 H, H-5), 3.44 (q, *J* = 5.9 Hz, 2 H, OCH_2_CH_2_CH_2_OH), 3.53 (t, *J* = 6.5 Hz, 2 H, OCH_2_CH_2_CH_2_OH), 4.42 (t, *J* = 5.0 Hz, 1 H, OH), 4.59 (s, 2 H, OCH_2_Ar), 7.03 (s, 1 H, Ar), 7.21–7.27 (m, 1 H, Ar), 7.31–7.38 (m, 6 H, Ar), 7.67 (m, 1 H, Ar), 15.16 (br s, 1 H, NH). ^13^C NMR (DMSO-d_6,_ 125 MHz) δ: 19.9 (CH_3_), 32.6 (OCH_2_CH_2_CH_2_OH), 36.0 (C-5), 45.3 (C-4/C-6), 46.5 (C-4/C-6), 57.6 (OCH_2_CH_2_CH_2_OH), 64.2 (OCH_2_Ar), 67.1 (OCH_2_CH_2_CH_2_OH), 106.3 (Ar), 108.8 (NH-C = C), 120.5, 121.1, 121.8, 123.6, 126.6, 126.8, 128.6, 129.5, 143.5, 148.2, 156.0 (Ar), 172.7 (NH-C = C). Analysis (calcd., found for C_26_H_27_NO_5_·2H_2_O): C (66.51, 66.11), H (6.66, 6.56), N (2.98, 3.28).

### Biological Methods

#### Compounds

In all these experiments, colchicine [obtained from Calbiochem (Darmstadt, Germany)] and CA-4P [fosbretulin, obtained from Sigma-Aldrich (Diegem, Belgium)] were added as reference compounds.

#### Cell lines

The human microvascular endothelial cell line HMEC-1 was obtained at passage 12 from the Centers for Disease Control and Prevention (Atlanta, GA, USA) and used from passage 17 till 27. Human breast carcinoma (MDA-MB-231), human cervical carcinoma (HeLa) and human T-lymphoid (CEM) cells were obtained from ATCC (Middlesex, UK). Cell lines were maintained in culture for up to 3 months and grown in DMEM, supplemented with 10% FBS, 0.01 M Hepes and 1 mM sodium pyruvate. All cells were cultured in a humidified 5% CO_2_ incubator at 37 °C.

### Cell proliferation

HMEC-1 cells were seeded in 48-well plates at 20,000 cells/well. After 24 h, 5-fold dilutions of the compounds were added. The cells were allowed to proliferate 4 days in the presence of the compounds, trypsinized, and counted by means of a Coulter counter (Analis, Belgium). HeLa and MDA-MB-231 cells were seeded in 48-well plates at 10,000 cells/well. After 24 h, different concentrations of the compounds were added. After 3 days of incubation, the cells were trypsinized and counted in a Coulter counter. Suspension cells (human lymphoid Cem cells) were seeded in 96-well plates at 60,000 cells/well in the presence of different concentrations of the compounds, allowed to proliferate for 96 h, and counted in a Coulter counter. The 50% inhibitory concentration (IC_50_) was defined as the compound concentration required to reduce cell proliferation by 50%.

### Cell cycle analysis

MDA-MB-231 cells were seeded in 6-well plates at 125,000 cells/well in DMEM with 10% FBS. After 24 h, the cells were exposed to different concentrations of the compounds. After 24 h, the DNA of the cells was stained with propidium iodide using the CycleTEST PLUS DNA Reagent Kit (BD Biosciences, San Jose, CA). The DNA content of the stained cells was assessed by flow cytometry on a FACSCalibur flow cytometer and analyzed with CellQuest software (BD Biosciences) within 3 h after staining. Cell debris and clumps were excluded from the analysis by appropriate dot plot gating. Percentages of sub-G1, G1, S, and G_2_/M cells were estimated using appropriate region markers^49^.

### Fluorescence detection of caspase-3 activity in live cells

MDA-MB-231 cells were seeded at 40,000 cells/cm². After 24 h, cells were incubated in Fluorobrite DMEM (Gibco), containing the compound and 2 µM of the caspase-3 substrate DEVD-NucView488 (Biotium, Hayward, CA). After 16 h, the DNA was stained with Hoechst33342 and the cells were imaged by fluorescence microscopy.

### Tube formation

Wells of a 96-well plate were coated with 70 µl matrigel (10 mg/ml, BD Biosciences, Heidelberg, Germany) at 4 °C. After gelatinization at 37 °C during 30 min, HMEC-1 cells were seeded at 60,000 cells/well on top of the matrigel in 200 µl DMEM containing 10% FCS. After 3 h of incubation at 37 °C, when the endothelial cells had reorganized to form tube-like structures, the compounds were added. Ninety minutes later, the cultures were photographed at 100 × magnification and analyzed using digitalized analysis.

### Cell migration

Wounds were created in confluent HMEC-1 cells by means of the “Incucyte® wound maker”. Next, different concentrations of compound and PI were added and images were taken every 2 min for the duration of the experiment. The algorithm masks each phase-contrast image to identify the position of the wounded (cell-free) and unwounded (cell-occupied) zones. Relative wound density is calculated and visualized in time-course plots.

### Determination of binding constants

#### Proteins and ligands

Calf brain tubulin was purified as described^50^. (*R*)-(+)-ethyl 5-amino 2-methyl-1,2-dihydro-3-phenylpyrido[3,4-b]pyrazin-7-yl carbamate (R-PT)^51^ was a kind gift of Prof. G.A. Rener, Organic Chemistry Research Department, Southern Research Institute, Birmingham, Alabama. The compounds were diluted in 99.8% D6-DMSO (Merck, Darmstadt, Germany) to a final concentration of 10 mM and stored at −80 °C.

#### Determination of binding constants

The binding constant of R-PT for dimeric tubulin was determined using the competition method in 10 mM sodium phosphate, 0.1 mM GTP pH 7.0 at 25 °C. To do so 0.2 µM of R-PT was incubated with increasing amounts of tubulin up to 10 µM and vice versa, 0.2 µM of tubulin was incubated with increasing amounts of R-PT up to 10 µM, the fluorescence emission spectra (excitation 374 nm) of the samples (5 nm excitation and emission slits) were determined using a Jobin-Ybon SPEX Fluoromax-2 (HORIBA, Ltd. Kyoto, Japan). Using these spectra it is possible to calculate the free and the bound R-PT concentration for each sample and thus to determine the binding constant of R-PT for tubulin.

Once K_b_ of R-PT is determined (5.1 × 10^6^ M^−1^) this compound could be used as a reference ligand as described in ref.^43^. For that purpose, the fluorescence emission of a previous mixed sample of 0.2 µM of R-PT and 0.2 µM of tubulin was evaluated in the presence of increasing concentrations of the studied ligand in a black 96-well plate (0; 0.05; 0.2; 0.5; 2; 5; 10; 30; 50; 70 µM). The samples were incubated 30 min. at 25 °C in a *Varioskan* plate reader (Thermo Scientific Waltham, Massachusetts, USA) before the fluorescence emission intensity at 456 nm (excitation 374 nm) was measured. The data were analyzed and the binding constants determined using Equigra V5.0^47^.

#### Determination of binding constant for compound **27**

In order to determine the binding constant of **27**, compound **22** (*K*_*b*_ = 9.1 × 10^7^ M^−1^) was used as a reference probe^47^ for a centrifugation-based method. In the experiment tubulin was incubated with over-stoichiometric ratios of both **22** and **27** and the bound (co-sediment with tubulin) and free (non co-sediment) concentrations of the compounds evaluated by HPLC. To ensure that the system has reached chemical equilibrium, both the displacement experiment of **22** for **27** and of **27** by **22** were performed. To do so, 200 μL of calf tubulin at 10 μM concentration were incubated with 15 μM of **27** or **22** for 45 min at 25 °C. Next, **22** or **27** were added and the incubation was maintained for additional 45 min under the same conditions.

Samples were transferred to a fixed-angle TLA-100 rotor (Beckman-Coulter) and centrifuged for 2 h at 100.000 rpm at 25 °C. Samples were divided, splitting the upper 100 μL in which only free compound is found from the lower, in which soluble tubulin remains with bound compounds. Appropriate controls were included to confirm the solubility of the compounds under the assay conditions. Fractions were extracted with 3 rounds of 1:1 volume of dichloromethane, concentrated by evaporation in SpeedVac and resuspended in 100 μL of acetonitrile.

To determine the concentration of the compounds, reverse-phase chromatography was performed using a SUPELCOSIL™ LC-18-DB HPLC Column coupled to an Agilent 1100 series HPLC system. Analytic separation of the compounds was performed in gradient, using as mobile phase CH_3_CN:H_2_O (35:65 to 70:30 in 30 min) obtaining well-defined peaks for **22** (retention time = 22 min) and **27** (retention time = 27 min) at λ = 317 nm.

Equilibrium *K*_b_ for **27** was determined by mass action calculations, using the following calculations:

The concentration of the compounds in the upper part of the tube was considered the free concentration in the experiment (*Cf*), the difference in the concentration of the compounds in the lower part of the tube with these in the upper part of the tube was considered the concentration of compound co-sedimenting with tubulin and thus the bound concentration in the experiment (*Cb*). Assuming the colchicine binding site to have a 1:1 stoichiometry:

1 $${{K}}_{{b}}{=}{\rm{C}}{\rm{b}}{/}{(}{\rm{C}}{\rm{f}}\times {\rm{T}}{\rm{f}}{)}$$

where *Tf* is the concentration of free tubulin, *Cf* and *Cb* are molar concentrations for free and tubulin-bound compound. Using simultaneous mass-action equation, we obtain the following expression^47^:

2 $${K}_{b27}={K}_{b22}\times [(C{f}_{22}/C{b}_{22})/(C{f}_{27}/C{b}_{27})]$$

allowing the determination of the binding constant *K*_*b*_ of **27** from the known *K*_*b*_ of compound **22** and the free and bound measured constants.

### X-Ray Crystallography

#### Crystallization, Data Collection, and Structure Solution

Crystals of T_2_R-TTL were generated as described^24–26^. Suitable T_2_R-TTL crystals were exchanged into reservoir solutions containing 0.5 mM TUB-015 or TUB-075 and soaked overnight. Soaked crystals were flash cooled in liquid nitrogen following a brief transfer into cryo solution containing 20% glycerol. T_2_R-TTL-TUB015 and T_2_R-TTL-TUB075 data were collected at beamline X06SA at the Swiss Light Source (Paul Scherrer Institut, Villigen, Switzerland). Images were indexed and processed using XDS^52^. Structure solution using the difference Fourier method and refinement were performed using PHENIX^53^. Model building was carried out iteratively using the Coot software^54^. Data collection and refinement statistics for T_2_R-TTL-TUB015 and T_2_R-TTL-TUB075 are given in Supplemental Table S1.

#### Structural Analysis and Figure Preparation

Molecular graphics and analyses were performed with PyMOL (The PyMOL Molecular Graphics System, Version 1.8.6.2. Schrödinger, LLC). Chains in the T_2_R-TTL complex were defined as follows: chain A, α-tubulin-1; chain B, β-tubulin-1; chain C, α-tubulin-2; chain D, β-tubulin-2; chain E, RB3; chain F, TTL.

### Computational Methods

#### Affinity maps calculation

Binding pocket analysis of the tubulin-TUB075 was carried out with cGRILL^33,34,55^, a computational tool formally equivalent to Goodford´s program GRID^37^. Hydrogen atoms, atom point charges and radii for all atoms in the complexes were calculated by submission to the H++ server (http://biophysics.cs.vt.edu) in order to obtain their pqr format files. Grid center was defined by selecting the corresponding ligand and a cubic box of 50 × 50 × 50 points and a grid spacing of 0.5 Å spacing was set for the calculations. cGRILL evaluates, at each grid point, the interaction energy between the whole receptor and five different probes combining van der Waals (Lennard-Jones potential), electrostatic (Coulombic), and geometry-based hydrogen bonding^56^ terms.

The five calculated affinity map, namely lipophilic (CH3), hydrogen bond acceptor (=O), hydrogen bond donor (NH4^+^), mixed hydrogen bond donor-aceptor (OH) and hydrophobic-like (hydrophobic), were visualized and analysed using the PyMOL plugin provided with the software.

Docking of compound 27. A three-dimensional cubic grid, consisting of 65 × 65 × 65 points with a spacing of 0.375 Å, was defined at the colchicine binding site in the α_2_β_2_ tubulin dimer from the ligand-free T_2_R-TTL-TUB075 complex. In agreement with recent proposal^35^, the side chain carboxylic group of βGlu200 was considered to be protonated. Electrostatic, desolvation, and affinity maps for the atom types present in 27 were calculated using AutoGrid 4.2.6 and then the Lamarckian genetic algorithm implemented in the automated docking program AutoDock4^57^. Intra- and intermolecular energy evaluation of each configuration allowed the selection of the 10 best-scoring solutions, which were virtually superimposable. The resulting [(α_2_:GTP:Mg^2+^)-(β_2_:27)] complex was then immersed in an octahedral solvent box extending 12 Å away from any protein or ligand atom and neutralized by addition of 31 sodium ions. All hydrogens and water molecules were first reoriented in the electric field of the complex and then 27, all protein residues, water molecules and counterions were subjected to 2 000 steps of steepest descent followed by 50 000 steps of conjugate gradient energy minimization. To improve the sampling, the resulting geometry-optimized coordinate set was used as input for a C_α_-restrained (5.0 kcal·mol^-1^Å^-2^) molecular dynamics (MD) simulation at 300 K and 1 atm using the GPU implementation of the *pmemd_cuda* engine in AMBER 14^58^. The standard ff14SB force field parameter set^59^ was used, which included updates for bioorganic phosphates and polyphosphates^60^, and the application of SHAKE to all bonds allowed an integration time step of 2 fs to be employed. The cutoff distance for the nonbonded interactions was fixed at 9 Å and the list of nonbonded pairs was updated every 25 steps. Periodic boundary conditions were applied and electrostatic interactions were represented using the smooth particle mesh Ewald method^61^ with a grid spacing of 1 Å. The coupling constants for the temperature and pressure baths were 1.0 and 0.2 ps, respectively. 500 sets of coordinates from the resulting MD trajectory were analyzed by means of the *cpptraj* module in AMBER^62^ and our in-house MM-ISMSA program^48^.

### PDB ID Codes

Coordinates have been deposited to the RCSB PDB (www.rcsb.org) under accession numbers 6FKJ (T2R-TTL-TUB075) and 6FKL (T2R-TTL-TUB015). Authors will release the atomic coordinates and experimental data upon article publication.

## Electronic supplementary material

Electronic Supplementary Material
