# Supplementary material for: High-affinity ligands of the colchicine domain in tubulin based on a structure-guided design
Source: Sci Rep. 2018 Mar 9;8:4242. doi: 10.1038/s41598-018-22382-x (PMC5844890; doi:10.1038/s41598-018-22382-x)
Supplement: Supplementary file 1 — Electronic Supplementary Material [file 41598_2018_22382_MOESM1_ESM.pdf]

Electronic Supplementary Information related to the article

High-affinity ligands of the colchicine domain in tubulin based on a structure-guided design

Oskia Bueno, Juan Estévez Gallego, Solange Martins, Andrea E. Prota, Federico Gago, Asier Gómez SanJuan, María-José Camarasa, Isabel Barasoain, Michel O. Steinmetz, J. Fernando Díaz, María-Jesús Pérez-Pérez, Sandra Liekens[c] and Eva-María Priego*

Includes:

Table S1. Data collection and refinement statistics

Figure S1. The T2R-TTL-TUB075 complex and ligand details

Figure S2. Established SAR for cyclohexanediones

References

Table S1. Data collection and refinement statistics.

	T_2R-TTL-TUB075	T_2R-TTL-TUB015
**Data collection**[a]		
Space group	$P2_12_12_1$	$P2_12_12_1$
Cell dimensions		
a, b, c (Å)	104.7, 157.5, 180.0	104.8, 157.8, 180.0
Resolution (Å)	49.45 – 2.15 (2.21 – 2.15)	49.44 – 2.10 (2.15 – 2.10)
R_{meas} (%)	11.8 (257.7)	11.4 (228.1)
R_{pim} (%)	3.4 (88.5)	3.3 (80.3)
$CC_{1/2}$[b]	99.9 (30.0)	99.9 (34.9)
$I/\sigma I$	16.5 (0.8)	16.7 (0.8)
Completeness (%)	99.5 (96.3)	98.5 (91.8)
Redundancy	12.5 (8.9)	12.3 (8.5)
**Refinement**		
Resolution (Å)	49.45 – 2.15	49.44 – 2.10
No. unique reflections	161351	171487
R_{work}/R_{free} (%)	17.5 / 20.6	17.8 / 21.7
Average B-factors ($Å^2$)		
Complex	60.7	57.0
Solvent	53.8	49.7
Ligand (chain B/D)	39.6 / 50.2	37.9/ 52.6
Wilson B-factor	42.5	39.7
Root mean square deviation from ideality		
Bond length (Å)	0.004	0.003
Bond angles (°)	0.719	0.613
Ramachandran statistics[c]		
Favored regions (%)	98.20	97.9
Allowed regions (%)	1.8	2.1
Outliers (%)	0	0

[a]Highest shell statistics are in parentheses. [b]CC1/2= percentage of correlation between intensities from random half-datasets[1] [c]As defined by MolProbity[2].

A

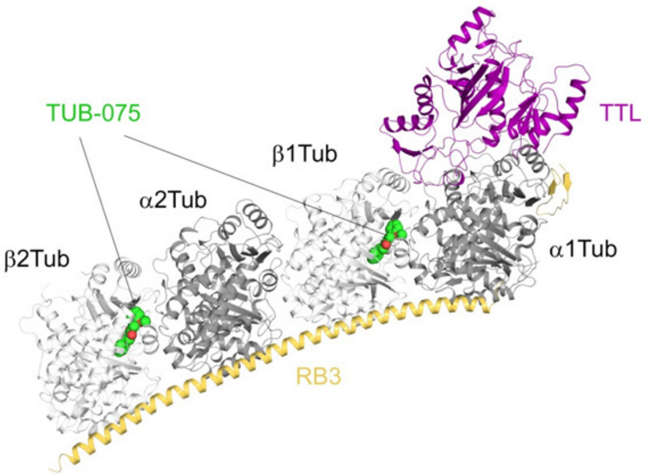

B

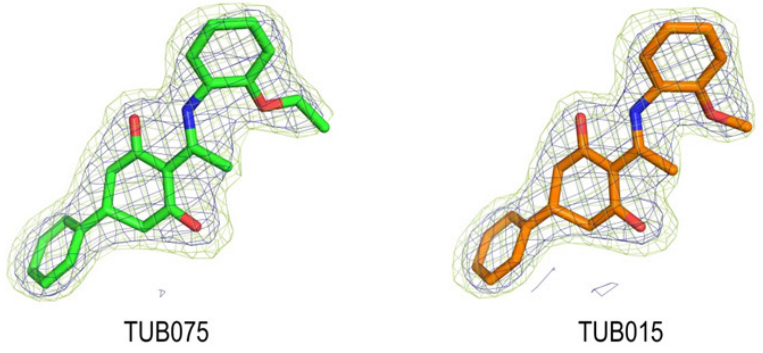

Figure S1. The T2R-TTL-TUB075 complex and ligand details. (**A**) Overall view of the T_2R-TTL-TUB075 complex structure. The α-tubulin and β-tubulin chains are in dark and light grey, TTL is in purple and RB3 is in yellow orange ribbon representation, respectively. The tubulin-bound TUB075 molecules are represented as green spheres, respectively. (**B**) Electron-density maps of both the TUB075 and TUB015 ligands bound to tubulin in their corresponding T_2R-TTL complexes. The SigmaA-weighted 2mFo - DFc (dark blue mesh) and mFo - DFc (light green mesh) omit maps are contoured at +1.0s and +3.0s, respectively. The map calculations excluded the atoms of the TUB ligands.

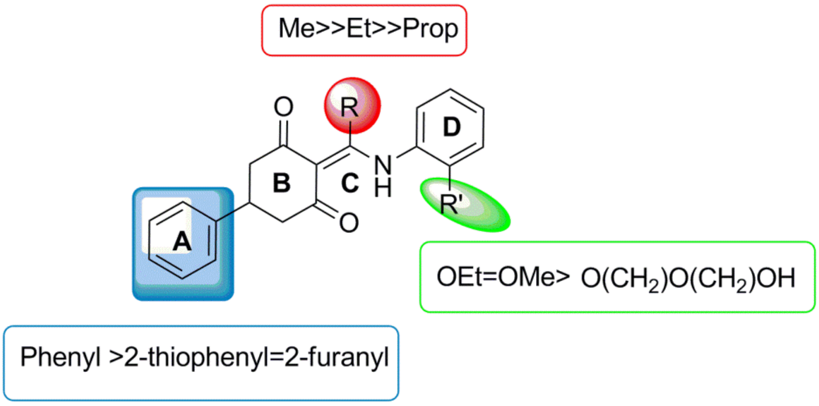

Figure S2. **Established SAR for cyclohexanediones**. Fragments of the cyclohexanedione derivatives and schematic representation of the most relevant structure-activity relationships established.[3,4]

References

1 Karplus, P. A. & Diederichs, K. Linking crystallographic model and data quality. *Science* **336**, 1030-1033, (2012).

2 Davis, I. W., Murray, L. W., Richardson, J. S. & Richardson, D. C. MOLPROBITY: structure validation and all-atom contact analysis for nucleic acids and their complexes. *Nucleic acids research* **32**, W615-619, (2004).

3 Canela, M. D. *et al.* Novel colchicine-site binders with a cyclohexanedione scaffold identified through a ligand-based virtual screening approach. *J. Med. Chem.* **57**, 3924-3938, (2014).

4 Canela, M. D. *et al.* Targeting the colchicine site in tubulin through cyclohexanedione derivatives. *RSC Adv.* **6**, 19492-19506, (2016).
